# Role of Resident Stem Cells in Vessel Formation and Arteriosclerosis

**DOI:** 10.1161/CIRCRESAHA.118.313058

**Published:** 2018-05-24

**Authors:** Li Zhang, Shirin Issa Bhaloo, Ting Chen, Bin Zhou, Qingbo Xu

**Affiliations:** 1 From the Department of Cardiology, the First Affiliated Hospital, School of Medicine, Zhejiang University, China (L.Z., T.C., Q.X.); 2 School of Cardiovascular Medicine and Sciences, King’s College London, BHF Centre, United Kingdom (S.I.B., Q.X.); 3 State Key Laboratory of Cell Biology, CAS Center for Excellence in Molecular Cell Science, Institute of Biochemistry and Cell Biology, Shanghai Institutes for Biological Sciences, University of Chinese Academy of Sciences, Chinese Academic of Sciences (B.Z.).

**Keywords:** cell lineage, myocytes, smooth muscle, neointima, stem cells, vascular diseases

## Abstract

Vascular, resident stem cells are present in all 3 layers of the vessel wall; they play a role in vascular formation under physiological conditions and in remodeling in pathological situations. Throughout development and adult early life, resident stem cells participate in vessel formation through vasculogenesis and angiogenesis. In adults, the vascular stem cells are mostly quiescent in their niches but can be activated in response to injury and participate in endothelial repair and smooth muscle cell accumulation to form neointima. However, delineation of the characteristics and of the migration and differentiation behaviors of these stem cells is an area of ongoing investigation. A set of genetic mouse models for cell lineage tracing has been developed to specifically address the nature of these cells and both migration and differentiation processes during physiological angiogenesis and in vascular diseases. This review summarizes the current knowledge on resident stem cells, which has become more defined and refined in vascular biology research, thus contributing to the development of new potential therapeutic strategies to promote endothelial regeneration and ameliorate vascular disease development.

During development, blood vessels are usually formed through 2 processes: vasculogenesis and angiogenesis. Vasculogenesis refers to the creation of the primary vascular network, as observed during embryonic development, where the blood vessels are formed de novo from progenitor cells (angioblasts).^1^ Angiogenesis consists of the sprouting of new blood vessels from the preexisting vascular networks and can occur at any time throughout an organism’s life. Vascular plasticity, either through vasculogenesis or angiogenesis, contributes to both vascular repair and vascular disease development.^2^ Accumulating studies have revealed that both vasculogenesis and angiogenesis require vascular progenitor cell homing or recruitment, proliferation, and differentiation into endothelial cells (ECs) and smooth muscle cells (SMCs), among other cell types.^3^ These vascular stem/progenitor cells play a key role in the formation of all types of vessels during development.

In adults, the arterial wall comprises 3 layers: tunica intima, which is the innermost layer consisting of a monolayer of ECs surrounded by a basal lamina and delimited by the internal elastic lamina; tunica media, the middle layer, which comprises predominantly SMCs supported by extracellular matrix proteins (elastic and collagen fibers); the adventitia, which is the external layer of the blood vessel containing a variety of cell types, including fibroblasts, macrophages, adipocytes, and pericytes. During the development of vascular diseases, it is believed that EC dysfunction/death induced by risk factors, for example, smoking and diabetes mellitus, could be an initiator that is followed by infiltration of inflammatory cells and deposition of oxidized lipids.^4,5^ In this process, some SMCs in the media can dedifferentiate and migrate into the intima together with inflammatory cells to form neointima.^6^ If the process occurs repeatedly, severe lesions can be developed, which could block the lumen of the vessels. Interestingly, recent studies indicate the presence of stem/progenitor cells in all 3 layers of the vessel wall^7,8^ (Table 1). Furthermore, these cells are shown to actively participate in endothelial repair/regeneration and in the formation of neointimal lesions, in response to injury.^43,44^ Therefore, the present article reviews the current state of research mainly focusing on the role of stem/progenitor cells in vessel formation during the embryonic stage and adult vascular remodeling during disease development, with emphasis on (1) the different origins and development of vascular progenitors during angiogenesis/vasculogenesis, (2) the mechanisms of stem cell differentiation into vascular lineages, and (3) the role of stem/progenitor cells in the pathogenesis of vascular diseases, including arteriosclerosis.

**Table 1.**
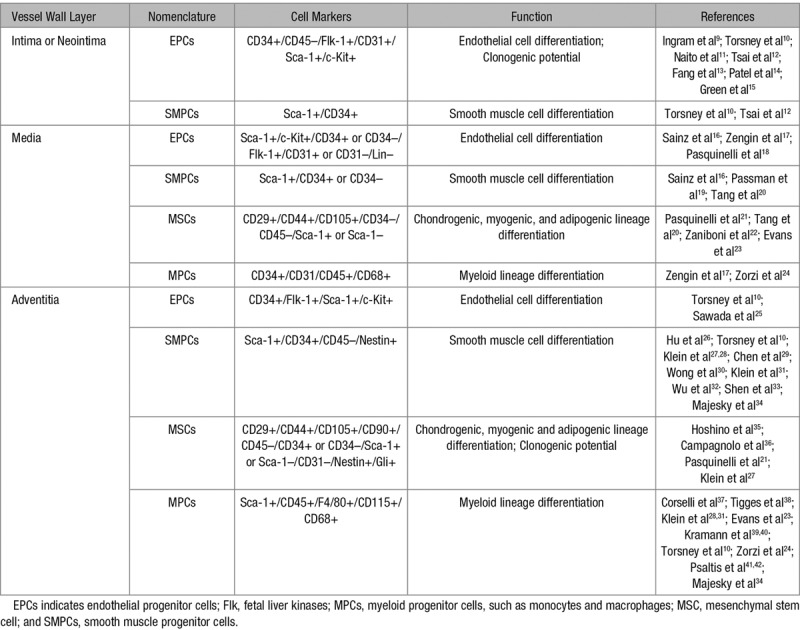
Vascular Stem/Progenitor Cells in the Vessel Wall

## Vascular Stem Cells in Development

Blood vessels arise from endothelial progenitors that share a common origin with hematopoietic precursors.^2^ These progenitors coalesce to form a primitive vascular labyrinth containing blood cells, and this primitive endothelial plexus progressively expands into a highly organized vascular network by means of vessel sprouting, pruning, and remodeling.^45^ In addition to supplying the developing organs with metabolic substances, blood vessels also provide a source of developmental signals during organogenesis and (later on in life) in tissue repair and regeneration.^46^ Vasculogenesis refers to the de novo formation of primitive vascular plexus from mesodermal progenitors, such as angioblasts.^3^ The term angioblast was first proposed a century ago by Sabin,^47^ who it in reference to a group of progenitor cells that form new blood vessels (outermost cells) and red blood cells (within the lumen or central cells). Conventional vasculogenesis is usually restricted to de novo blood vessel formation during embryonic development.^48^ After vasculogenesis at the initial stage, further blood vessels are generated through angiogenesis, which refers to the generation of a new blood vessel through sprouting or splitting of preexisting vessels, which is followed subsequently by pruning and remodeling into a functional adult circulatory system.^45^

### Multipotent Vascular Stem Cells: Mesoangioblasts

**Figure 1.**
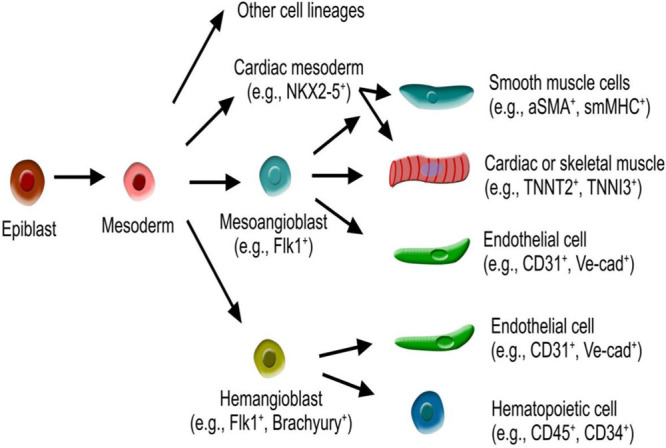
**Schematic figure showing mesoangioblast and hemangioblast-derived from mesoderm.** Mesoangioblasts are multipotent and give rise to endothelial cells, cardiac or skeletal muscle, and smooth muscle cells. Hemangioblasts are bipotent and give rise to endothelial cells and hematopoietic cells. aSMA indicates smooth muscle actin-α; smMHC, smooth muscle myosin heavy chain; TNNI, troponin I; TNNT, troponin T; and Ve-cad, VE-cadherin.

Mesoangioblasts are multipotent progenitors of mesodermal tissues that contribute to both vascular and extravascular mesodermal lineages in development and are marked by high expression of VEGFR2/Flk1 (vascular endothelial growth factor receptor/fetal liver kinases).^49^ The mesoangioblasts originate from the dorsal aorta and differentiate into most mesodermal tissues.^50^ By quail-chick engraftment experiments, Minasi et al^50^ showed that aortic cells differentiate into vascular cells and also into other mesodermal lineages, such as blood, cartilage, bone, smooth, skeletal, and cardiac muscle. These vessel-associated stem cells, the mesoangioblasts, participate in postembryonic development of the mesoderm, representing an important origin of vascular stem cells.^50^ Interestingly, some ECs isolated from embryonic vessels can differentiate into beating cardiomyocytes and express cardiac markers both in vivo and in vitro.^51^ An elegant in vivo study using replication-defective retroviral infection showed that myogenic cells and ECs are derived from a common somatic precursor that resides in the paraxial mesoderm.^52^ In addition to ECs, SMCs of the dorsal aorta share a common clonal origin with skeletal muscle during development.^53^ By comparison, coronary artery SMCs share a common origin with cardiomyocytes, both of which are derived from Nkx2-5^+^ progenitors^54^ that originate from cardiac mesoderm. Another study using Flk1 lineage tracing showed that Flk1^+^ cells are progenitors for muscle lineages and hematopoietic and vascular ECs.^55^ It remains to be determined whether single Flk1^+^ progenitors give rise to both muscle and vascular lineages for clonal analysis would be crucial. These above-mentioned pioneering studies documented that mesoangioblasts constitute a subset of vascular stem/progenitor cells during development (Figure 1). Furthermore, transplantation of mesangioblast-like, vessel-associated stem cells corrected the dystrophic phenotype of a dystrophic mice model, morphologically and functionally. Part of the therapeutic effect was because of the widespread distribution of the transplanted mesoangioblasts throughout the capillary network.^56^ Hence, identification of mesoangioblasts not only uncovers an unexpected source of progenitors for skeletal or cardiac muscle and a variety of other mesoderm-derived tissues but also establishes a lineage relationship between precursors of vascular and extravascular mesodermal tissues during development. The new insights have important implications for the study of stem cell biology and regenerative medicine.^57^

### Bipotent Vascular Stem Cells: Hemangioblasts

In early embryonic development, hematopoiesis and vasculogenesis are coupled in the extraembryonic yolk sac, where primitive ECs are closely associated with hematopoietic cell lineages. The observation that hematopoietic cells and angioblasts arise in close proximity has long fuelled speculation that a common precursor, the hemangioblast, may exist as the progenitor for both blood and the vascular system^47^ (Figure 1). Using embryonic stem cell–derived embryonic bodies, Choi et al^58^ identified a common precursor for hematopoietic and ECs. A subsequent study showed that hemangioblasts are a subpopulation of mesodermal cells that expresses Flk1 and brachyury and whose hematopoietic and vascular commitment is initiated in the primitive streak of the mouse embryo.^59^ Through the construction of single cell-resolution fate maps, Vogeli et al^60^ confirmed that individual bipotent cells can give rise to both hematopoietic and ECs. However, other studies suggested that the hematopoietic and the EC lineages are independently fated among mesodermal progenitors during gastrulation.^61,62^ Lineage tracing studies and clonal analysis of mouse development revealed that hematopoietic and EC lineages are segregated and independent.^63^ The debate over the existence of bipotent hemangioblasts has not been completely resolved, and more advanced genetic lineage tracing methods combined with single cell sequencing technology may help to elucidate this important issue in the future. Irrespective of this controversy, knockout of Flk1 resulted in abnormal blood islands and vasculogenesis, indicating that VEGF signaling governs the differentiation and proliferation of both lineages.^64,65^ An additional study also demonstrated that Flk1 is required for both hematopoietic and EC development.^66^

### Smooth Muscle Progenitors

Perivascular mural cells are comprised 2 cell types that are required to stabilize vascular networks: pericytes and SMCs. Pericytes surround smaller caliber vessels whereas vascular SMCs surround larger vessels.^67^ Here, we focus on the recent advances in our understanding of the developmental origin of organ-specific pericytes and SMCs; and use aorta and heart tissues as examples to introduce the heterogeneity of their developmental origins. We also include in this section discussion of the controversy over whether pericytes can be endogenous multipotent stem cells.

**Figure 2.**
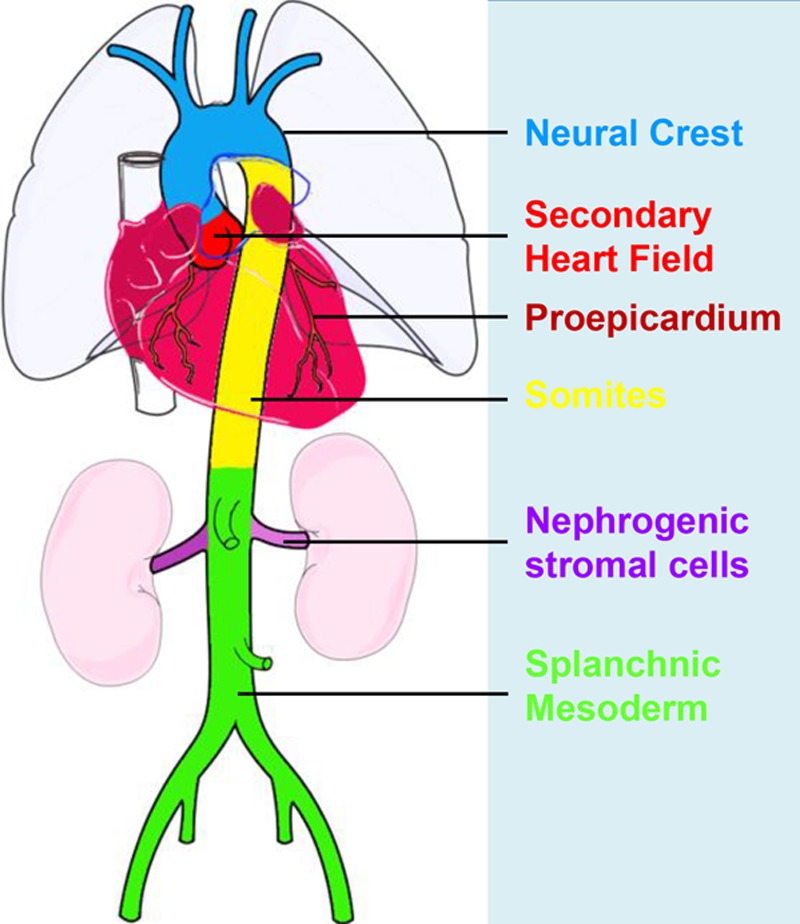
**Developmental fate map of vascular smooth muscle.** The different colors represent the different embryonic origins for smooth muscle cells (SMCs), as indicated in the figure. Aortic SMCs originate from 3 distinct developmental lineages. The secondary heart field (red) contributes to SMCs of the basal aortic root, the neural crest (blue) gives rise to SMCs of the ascending aorta and aortic arch, the paraxial/somitic mesoderm (yellow) contributes to SMCs of the descending aorta. The lineage boundaries depicted are shown to approximation and may change with aging.

One of the hallmarks of arterial SMCs is the heterogeneity of their embryological origin, which may significantly influence the development of vascular diseases. For example, there are 3 distinct origins for SMCs in different segments of the aorta. The Isl1^+^ secondary heart field contributes to SMCs of the basal aortic root^68,69^; Wnt1^+^ neural crest cells contribute to SMCs of ascending aorta and the aortic arch^70^; Meox1^+^ cells of paraxial mesoderm contribute to SMCs of the descending aorta.^71^ The diversity of the developmental origins may, in part, contribute to the site-specific location of vascular diseases because of different properties of the SMCs in each segment, such as gene expression, protease profiles, and cell signaling pathways.^25^ For more detailed information, a recent review on SMC origins and differentiation has been published^72^ and is summarized in Figure 2. Interesting functional differences have been detected between SMCs of different embryological origins. For example, when exposed to TGFβ (transforming growth factor-beta) signaling, collagen production is increased in SMCs from neural crest origin, but not from mesodermal zone. Homocysteine, a protein related to cardiovascular disease, has been shown to stimulate proliferation of SMCs from neural crest but not from mesodermal origin.^73^ Detailed pathophysiological studies of each segment have identified disparities in atherosclerotic plaque deposition, aortic aneurysm distribution, and vascular calcification.^74^ Because of these differences, it is important to dissect the pathophysiological roles of each segmental SMC population, specifically in the development of diseases. Generation of human vascular smooth muscle subtypes, by defined differentiation of human pluripotent stem cells, provides a new in vitro means to model origin-dependent disease susceptibility and to develop bioengineered vascular grafts for regenerative medicine.^75^ Improved genetic tools would be valuable to precisely target each segmental SMC subtype to understand their distinct in vivo roles in multiple diseases.

Distinct from large arteries, SMCs in most organs are derived from mesothelial cells that cover visceral organs and tissues.^76^ Lineage tracing studies showed that these Wt1^+^ mesothelial cells are progenitors of SMCs or pericytes in the organs, such as intestine, lung, and heart. In the developing heart, coronary SMCs originate from the proepicardium, a structure that sits between the atrioventricular groove and the septum transversum.^77^ Mesothelial cells migrate from the proepicardium and cover the developing heart to form a single layer of epicardial cells.^70^ In response to inducing signals from the underlying myocardium, epicardial cells delaminate from the epicardium and undergo an epithelial-to-mesenchymal transition to form mesenchymal cells.^78^ A subset of epicardium-derived cells forms pericytes and SMCs of coronary vessels and also cardiac fibroblasts.^76,79^ The migration and differentiation of epicardial cells to generate SMCs are tightly regulated by several signaling pathways, including TGFβ, retinoic acid, Notch, PDGF (platelet-derived growth factor), FGF (fibroblast growth factor), and Wnt/β-catenin.^80–83^ A recent study shows that these coronary SMCs derive from pericytes, in which Jag1-Notch3 signaling regulates their differentiation.^84^ Furthermore, endocardial cells, from the innermost layer of the myocardium, also undergo epithelial-to-mesenchymal transition to form mesenchymal cells, and some of them contribute to pericytes and SMCs.^79,85^ The differentiation of endocardial cells to coronary SMCs requires canonical Wnt signaling involving Frizzled4, β-catenin, and EC-derived Wnt ligands.^85^ In the developing heart, endocardial and epicardial progenitors coordinately contribute to the majority of coronary SMCs.^69^ Whether adult endocardial or epicardial cells continue to contribute to SMCs under homeostasis or after injury remains largely unexplored.^86^

### Bipotent Progenitors of Endothelial and SMCs

ECs and SMCs originate from different progenitors in most organs and tissues. However, there are some conditions under which a common progenitor exists for these 2 distinct cell populations. Flk1^+^ cells derived from embryonic stem cells differentiate into both ECs and mural cells in vitro and in vivo, indicating Flk1^+^ cells as bipotent vascular progenitors.^87^ In mouse tissue, ECs marked by Nfatc1 contribute to both vascular ECs and a subset of pericytes during development,^85^ implying a bipotent potential of Nfatc1^+^ cells for both vascular lineages. In the developing heart, Isl1^+^ and Flk1^+^ cardiac stem cells contribute to both ECs and SMCs, in addition to cardiomyocytes.^88,89^ In the developing heart, Gata4^+^ or Wt1^+^ epicardial cells also contribute to both coronary ECs and SMCs.^90^ In the adult tissue, protein C receptor–expressing cells serve as bipotent endogenous vascular endothelial stem cells that give rise to de novo formation of ECs and pericytes.^91^ However, it remains equally possible that ECs from protein C receptor–expressing cell lineages undergo endothelial-to-mesenchymal transition and differentiate into SMCs. The bipotency of these cells needs further rigorous investigation with improved technology in the future. Furthermore, the molecular mechanisms that determine the diversification of endothelial and SMC lineages from a bipotent progenitor remain to be elucidated.

## Vascular Resident Stem Cells in Adults

In 2001, Alessandri et al,^92^ by performing an aortic ring assay with human embryonic aortas, showed ex vivo outgrowth of capillary-like structures. Moreover, the cells isolated from these outgrowths were CD34^+^/CD31^−^ and differentiated in vitro into to ECs.^92^ This suggests that the vessel wall itself could contain resident vascular progenitor cells. In 2003, Majka et al^93^ isolated vascular progenitor cells resident in adult skeletal muscle, which could be engrafted and differentiated into the endothelium and smooth muscle. In the same year, Tintut et al^94^ also observed that the artery wall contains cells resembling mesenchymal stem cells (MSCs), with the ability to demonstrate multipotency in vitro. In 2004, Hu et al^26^ provided the first evidence of vascular progenitor cells resident in the adventitia layer of an adult vessel wall. These adventitial progenitors expressed the markers stem cell antigen-1 (Sca-1), c-Kit, CD34, and Flk1. Among these, the adventitial Sca-1^+^ cells were able to differentiate in vivo into SMCs and to participate in neointima formation.

Since then, numerous studies^13,15,20–24,27,28,30–33,35,37,38,95–106^ have been published proving the presence of vascular progenitor cells resident in the vessel wall as a reservoir for multipotent stem/progenitor cells (Tables 1 and 2). For instance, Ingram et al^9^ showed that endothelial progenitor cells, resident in the intima of umbilical vein and aortic vessels, could contribute to EC formation. MSCs were also detected in the inner surface of human varicose saphenous vein. These cells were able to differentiate in vitro into osteoblasts, chondrocytes, and adipocytes.^108^ Also in human saphenous vein, Campagnolo et al^36^ observed the localization of CD34^+^ cells around the adventitial vasa vasorum that showed clonogenic and multilineage differentiation capacities. Zengin et al^17^ reported that CD34^+^/CD31^−^ progenitor cells with the ability to differentiate into mature ECs, hematopoietic cells and macrophages, were present in a region contained between the media and the adventitia of adult human blood vessels. Pasquinelli et al^18^ demonstrated the presence of angiogenic MSCs within the human thoracic aorta. Still in the media layer, Sainz et al^16^ isolated a side population of progenitor cells displaying a Sca-1^+^/c-Kit^(−^^/low)^/Lin^−^/CD34^(^^−/low)^ profile, which generated cells with EC and SMC phenotypes that could form vascular-like branching structures. Furthermore, Torsney et al^10^ identified progenitor cells expressing Sca-1, CD34, and c-Kit markers within the neointima and adventitia of human atherosclerotic arteries, suggesting that these cells could be the source of SMCs, ECs, and macrophages that form atherosclerotic lesions. Pericytes, which are normally regarded as mural cells, have also been described to possess multipotent stem/progenitor cell properties although controversy still exists on their distinction from MSCs.^109,110^

**Table 2.**
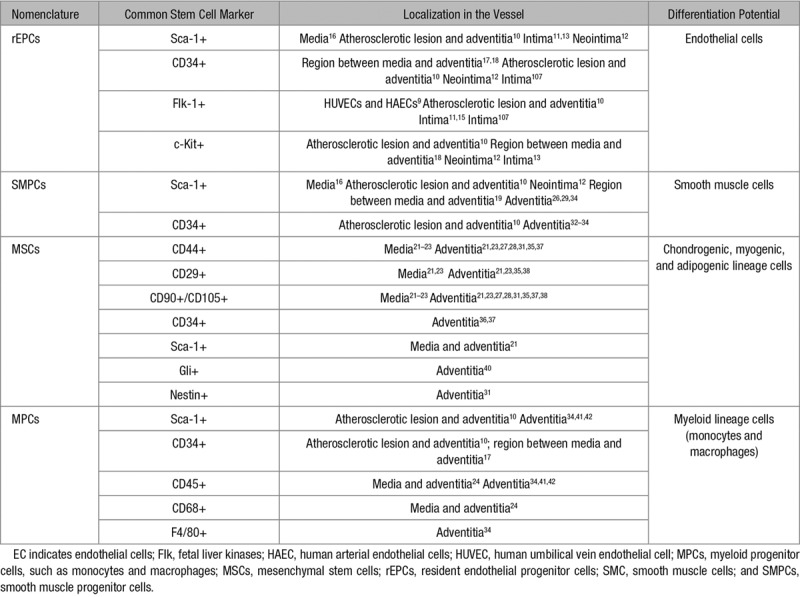
Differentiation Potential of Vascular Resident Stem/Progenitor Cells

### Resident Endothelial Progenitors

**Figure 3.**
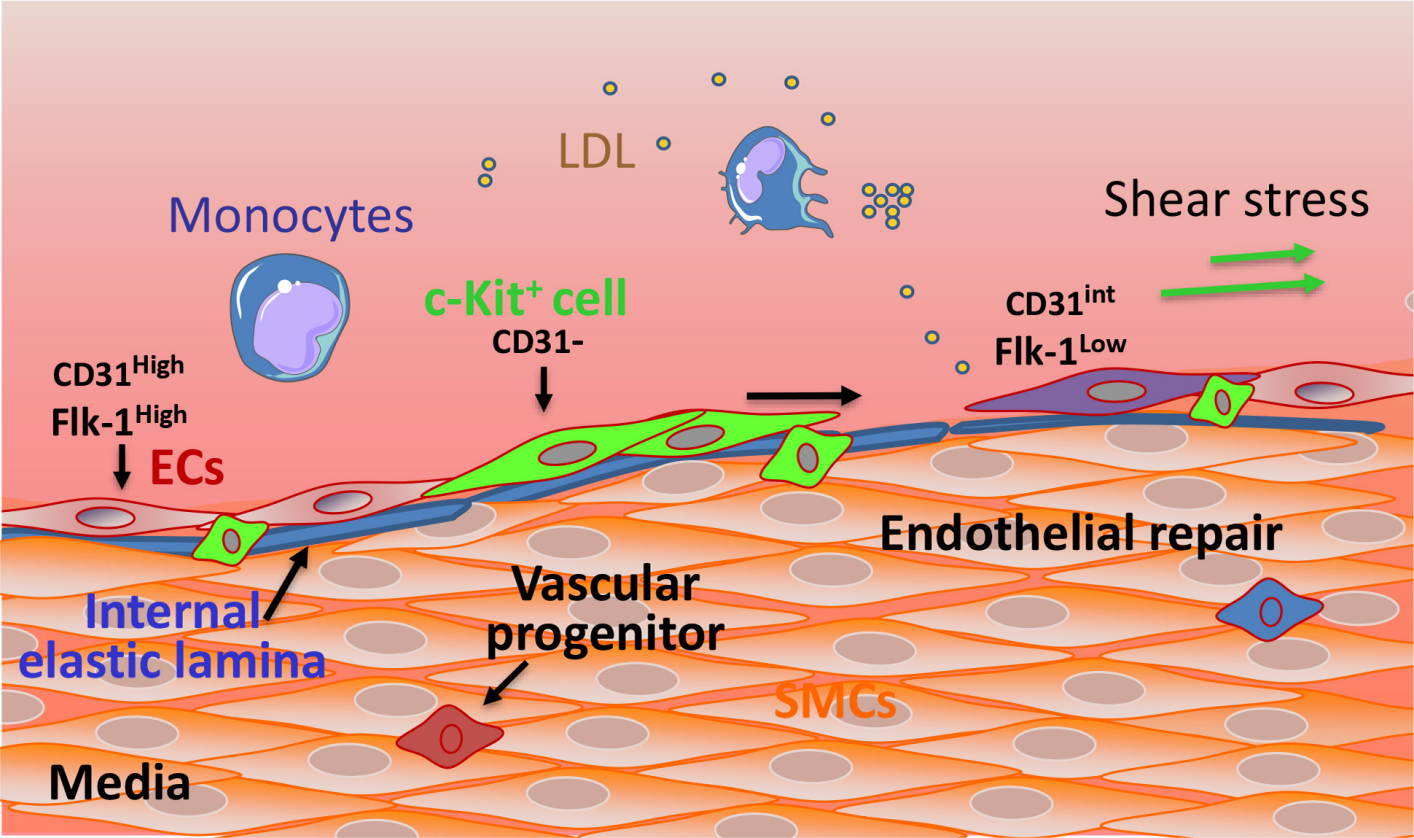
**Proposed roles for vascular wall resident endothelial progenitors in endothelial generation.** The vessel wall comprises an inner layer (intima), a thick media layer, and an outer layer (adventitia). The intima is composed of the elastic lamina (blue) and a monolayer of endothelial cells (ECs). The intima encompasses mature and terminally differentiated cells (ECs: CD31^High^/Flk-1^High^) and also a niche of endothelial progenitors, such as c-Kit^+^ cells (green), which can proliferate, migrate, and differentiate into ECs. Endothelial progenitors (EPCs) have clonogenic and endothelial generative potential and express stem cell markers, such as c-Kit, associated with low expression of CD31. EPCs are progenitors of a transient amplifying population of cells (purple) which display an intermediate expression of CD31 and a low expression of Flk-1. In response to EC turnover and under regulatory signals, the endothelial progenitors can be activated and undergo migration, proliferation, and differentiation to replace lost ECs. Flk indicates fetal liver kinases; LDL, low-density lipoprotein; and SMC, smooth muscle cell.

In the small intestine paradigm, stem cells in the crypt give rise to transient amplifying cells, which proliferate and further differentiate into enterocytes in the villi.^111^ Whether there is a similar hierarchy when preexisting ECs generate new ECs during angiogenesis remains poorly understood. Using human umbilical vein ECs and human aortic ECs, Ingram et al^9^ demonstrated that a hierarchy of endothelial progenitor cells can be identified and discriminated by their clonogenic and proliferative potential. By a Hoechst dye exclusion method, stem cells in the luminal surface of preexisting blood vessels have also been identified as an EC side population. This population is dormant in the steady state but generates a significant number of ECs when transplanted into ischemic regions and restores blood flow of ischemic tissue.^11^ The notion of the presence of resident endothelial progenitor cells with clonal proliferative and self-renewal capacities in the endothelium lining of the vessels has been widely discussed by Yoder et al,^9,112,113^ who have provided solid evidence in their studies. Recent work found that there are actually 3 subpopulations of ECs defined by their expression of CD31, VE-cadherin, Sox18, and Flk1. Lineage tracing studies demonstrated that CD31^−^/Flk1^lo^ is an endovascular progenitor, CD31^int^/Flk1^lo^ is transit amplifying, and CD31^hi^/Flk1^hi^ is a differentiated cell population.^14^ The stepwise differentiation from endothelial progenitors to transit amplifying to mature ECs is governed by Sox18/SoxF transcriptional regulation.^14^ In addition, a single c-Kit^+^ adult vascular endothelial progenitor cell is capable of undergoing clonal expansion to generate millions of endothelial daughter cells in vitro and functional blood vessels that connect to host circulation.^13^ Lineage tracing studies on c-Kit^+^ cells revealed that a subset of ECs express this stem cell marker^114–116^ (Figure 3). Whether the endogenous c-Kit^+^ cells are resident vascular progenitors and have the potential to generate new blood vessels after injury remain to be investigated. Given the difficulty from the previous literature cited above to exactly define endothelial progenitors in terms of nomenclature or immunophenotyping, Yoder et al^107,113,117^ have provided important guidelines for precise terminology. Notwithstanding, the identification of EC hierarchy shifts our understanding of vascular resident endothelial progenitors and their role in tissue repair and regeneration, opening new avenues for better understanding and improved treatment of cardiovascular diseases.

### Sca-1^+^ Vascular Progenitor Cells

The Sca-1 marker, also known as Ly-6A/E, was originally used to define adult murine hematopoietic stem cells.^118^ Under homeostatic conditions, the endothelium of large arteries exhibits little Sca-1^+^ expression. However, in response to injury, the number of Sca-1^+^ cells in the endothelial layer of the aorta increases.^119^ In addition, apolipoprotein E–deficient (ApoE^−/−^) mice, which model atherosclerosis, have elevated numbers of Sca-1^+^ cells in the aortic endothelium.^120^ Hu et al^26^ demonstrated that adventitia-resident vascular progenitor cells express Sca-1 and that these cells contributed to atherosclerosis via migration and differentiation into SMCs. Among other studies, the work of Yu et al^121^ further supported the notion that resident Sca-1^+^ cells contribute to neointima formation, which may increase plaque burden. Nevertheless, Sca-1^+^ cells may also harbor a potential for reparative and regenerative processes. The differentiation of Sca-1^+^ cells into SMCs or ECs may participate in endothelial layer regeneration^122^ and promote atherosclerotic plaque stability.^123^ Furthermore, this capacity of Sca-1^+^ cells could find a useful application of in tissue engineering, such as artificial vessel generation.^124^

### Mesenchymal Stem Cells

MSCs are a group of multipotent stromal cells that can differentiate into various cell types, including osteoblasts, chondrocytes, and adipocytes.^125^ MSCs can be isolated from several different tissues, including umbilical cord, bone marrow, and adipose tissue.^126^ Their properties vary from tissue to tissue. For instance, cells isolated from adult bone marrow display a stable phenotype, expressing SH2, SH3, CD29, CD44, CD71, CD90, CD106, CD120a, CD124, and many other surface proteins.^127^ Understanding the mechanisms driving cell differentiation, migration, mobilization, and homing will contribute to future clinical application of these cells as treatments for vascular diseases. Because of their easy isolation and relative abundance compared with other types of MSCs, adipose tissue stem cells have recently become popular in stem cell research.^128^ The stromal vascular fraction can be simply obtained by collagenase digestion of adipose tissue and centrifugation to remove mature adipocytes by floatation. Stem cells isolated from human subcutaneous adipose tissue display a specific phenotype, CD34^+^, CD13^+^, CD45^–^, CD14^–^, CD144^–^, and CD31^–^,^129^ and can give rise to adipocytes or ECs in vitro. Adipose-derived stem cells that express Sca-1^+^, CD34^+^, CD45^–^, and CD31^+^^130^ can differentiate into white adipose tissue in mice. One report also demonstrated that CD34^+^ adipose-derived MSCs could be maintained for 20 weeks^131^ although CD34^−^ adipose-derived stem cells are more plastic.^132^

Although cell culture provides a good approach for studying differentiation, migration, and proliferation in vitro, it is more relevant to study the adipose-derived stem cells in their biological context in vivo. For example, injection of allogeneic abdominal adipose-derived stem cells, either with or without transfection with plasmid-VEGF165, provided a protective role in a rabbit model of critical hindlimb ischemia. They increased arteriolar density and protected against ischemia-induced muscle lesions.^133^ Furthermore, Joe et al^134^ purified Lin^−^/Sca-1^+^/CD34^+^ cells from the stromal vascular fraction of white adipose tissue of GFP (green fluorescence protein) mice by fluorescence-activated cell sorter and transplanted them in matrigel into wild-type mice. Adipocyte progenitor cells were able to form mature unilocular and multilocular adipocytes with GFP expression. In other research, Miranville et al^135^ reported that CD31^−^/CD34^+^ stromal vascular fraction cells can differentiate into ECs that express CD31 and develop vascular structures in Matrigel in response to VEGF. Hence, adipose tissue–derived stem cells may participate in angiogenic processes.

### Pericytes as Endogenous Multipotent Stem Cells

Cultured pericytes isolated from distinct tissues can also differentiate into multiple cell types, including osteogenic, chondrogenic, and adipogenic lineages.^136^ Hence, perivascular cells, principally pericytes, appear to harbor a reservoir of progenitor cells that are an endogenous source of MSCs. In vivo lineage tracing studies using a PDGFRβ (platelet-derived growth factor receptor-beta)-Cre line showed that the mural cell compartment of the adipose vasculature contains progenitors of white adipocytes.^130^ Furthermore, pericytes have also been reported to give rise to follicular dendritic cells^137^ and skeletal myocytes.^138^ Guimaraes-Camboa et al^139^ used inducible Tbx18-CreERT2 line to permanently label pericytes and vascular SMCs of multiple organs in vivo and to follow their fate in aging and injury models. Surprisingly, these labeled pericytes remained as pericytes and did not behave as stem cells by differentiating into other cell lineages, challenging the current view of pericytes as tissue-resident MSCs. There are several possible explanations for these discrepant findings. First, the differentiation potential of transplanted cells can be influenced by ex vivo manipulations and hence may not be equivalent to authentic in vivo tracing studies.^140^ Second, the differences in the lineage tracing results observed between previous studies and Evan’s could be because of the constitutive PDGFRβ-Cre line used in the former studies.^130,137^ PDGFRβ could be expressed in multiple cell lineages, rendering its promoter unsuitable for tracking the progeny of pericytes. By comparison, Evan’s Tbx18-CreERT2 lineage tracing marker^139^ is inducible during development and the Cre activity is, therefore, more controlled temporarily. Moreover, the discrimination of perivascular cells, such as between pericytes or fibroblasts, can be challenging if the tracing is dependent on a single marker. For example, a subset of fibroblasts expresses PDGFRβ and certain populations of pericytes express PDGFRα.^141^ Obviously, the previous studies rely on currently known gene profiles that we understand identify pericytes. With the advent of single cell sequencing and improved genetic lineage tracing technology, a more complete understanding of endogenous perivascular stem cells could be achieved.

## Vascular Progenitor Cells in Arteriosclerosis

Arteriosclerosis includes native (spontaneous) atherosclerosis, transplant arteriosclerosis, angioplasty-induced restenosis, and vein graft arteriosclerosis.^142,143^ For atherosclerosis, it is believed that a combination of lipid and flow-mediated endothelial dysfunction/death initiates an inflammatory response followed by SMC migration and proliferation to form neointimal lesions.^144^ Consequently, focal atherosclerotic plaques are formed, containing a necrotic core encompassing lipid deposition, SMCs, leukocytes—mainly macrophages—and associated debris. Endothelial dysfunction and inflammation also contribute to the other types of arteriosclerosis. Today, many studies demonstrate that vascular progenitor cells can contribute to either endothelial repair, probably beneficial, or proliferative processes that might increase the severity of the arteriosclerotic lesion. Considering the body of work published in the literature and summarized in Table 1 on the presence of vascular progenitor cells in the vessel wall, these cells are a potential source of ECs and SMCs in the development of arteriosclerosis.

### Effect of Risk Factors for Atherosclerosis on Resident Stem Cells

Genetic or environmental risk factors that lead to hypertension, hyperlipidemia, hyperglycemia, systemic inflammation, circulation of reactive oxygen species (ROS), among other conditions can cause endothelial injury and dysfunction. Smoking is one of the major causes of cardiovascular disease because it induces hypertension, endothelial dysfunction, leukocyte activation, and generation of ROS, which decrease nitric oxide availability.^145^ ROS are toxic biproducts that lead to oxidative stress, which can activate important transduction pathways, such as G protein–coupled and growth factor receptors, Notch, and Wnt signaling. As a result, vascular cell, including stem/progenitor cell, migration, proliferation, and differentiation are affected. For example, TGF-β, an SMC differentiation factor, activates Nox4 which produces ROS. These in turn upregulate the expression of serum response factor and hence activate *SMC* gene transcription.^146^ ROS also induce MSC proliferation and progenitor migration and proliferation by activating MAPKs (mitogen-activated protein kinase) and PI3K/AKT (phosphatidylinositol-3-kinases/protein kinase B) pathways.^147,148^ Hypertension also induces oxidative stress on the arterial wall and vascular remodeling. Areas of the vessel with disturbed flow reveal higher ROS accumulation and endothelial turnover that can culminate in lesion formation. Vascular stem cells may be mobilized and undergo differentiation to try to repair the damaged vessel. For instance, Xiao et al^149^ demonstrated that Sca-1^+^ progenitor cells could differentiate into endothelial lineage in response to laminar flow or VEGF treatment while the flow stimulation in vitro suppressed smooth muscle lineage differentiation. Similarly, laminar flow increased Flk1^+^ cell proliferation and differentiation toward ECs when compared with static controls.^150^ Hypertensive vessels possess characteristic intimal thickening and medial hyperplasia, associated with enhanced expression of α-smooth muscle actin. These results suggest that stem/progenitor cell migration, proliferation, and differentiation could be a response to cytokines released by the injured endothelium and the underlying inflammation process. Furthermore, mechanical stretching via PDGFR/Ras/ERK (extracellular signal–regulated kinase) pathway is reported to result in the differentiation of progenitors into SMCs.^151^ For example, in pulmonary artery hypertension, resident PW1^+^ progenitor cells undergo proliferation and differentiation into SMCs induced by chronic hypoxia, via CXCR4 (C-X-C chemokine receptor) activation.^152^ Interestingly, hypoxia promotes the proliferation of resident progenitors in the lung.^153^ Diabetes mellitus–associated vascular diseases show hypoxic tissue, which may be a stimulus for resident progenitors, but there is also hyperglycemia and oxidative stress, endothelial dysfunction, and vessel wall remodeling. With increased levels of ROS and decreased nitric oxide, progenitor mobilization is hampered, and under hyperglycaemia, glycosylated proteins induce progenitor senescence via activation of AKT/p53/p21.^154^ Hence, there may be a stimulus for expansion progenitors that is frustrated by other factors. Diabetes mellitus and obesity are risk factors for atherosclerosis, and obesity-related increased perivascular adipose tissue is increasingly accepted to play an active role in vascular remodeling. Adipocytes secrete adipokines and cytokines, which may act not only in an autocrine way but also on resident stem cells in the vessel. Recently, Xie et al^155^ revealed that leptin induces the migration of adventitia-derived Sca-1+ progenitor cells after vessel injury, contributing to neointima lesion formation (Figure 5). However, the effect of leptin and other adipokines on resident vascular progenitor cells, particularly in the context of diabetes mellitus– or obesity-induced vascular diseases, remains to be fully clarified.

### Endothelial Repair and Regeneration

As mentioned above, a key initiating event of arteriosclerosis is the dysfunction/death of the endothelial layer, with increased permeability and impaired endothelium-mediated vasodilation. Endothelial turnover is an important compensatory mechanism but also contributes to endothelial leakiness and dysfunction. ECs in the areas of the artery resistant to atherosclerosis have a lifespan of ≈12 months in rats, whereas cells at lesion-prone sites live for weeks and for even shorter times when animals are aged.^156^ A study from our group demonstrated that the numbers of dying/proliferating cells in the aortas of ApoE^−^^/−^ mice and of wild-type animals were significantly different.^119^ Importantly, lesion-prone areas displayed a higher turnover rate of ECs, as indicated by BrdU (bromodeoxyuridine)-positive labeling. However, the endothelium covering early lesions was leaky because of lower levels of tight junctions.^119^ The question is, whether these highly proliferative, leaky ECs are derived from mature ECs or from a specific population of vascular progenitors (see Figure 3). Interestingly, endothelial progenitors that are resident in the intima and shown to participate in vessel formation have higher proliferative ability and increased expression of matrix metalloproteases and growth factor signaling, all of which would favor their participation in endothelial regeneration.^14^ But why do ECs in certain regions display increased turnover and loss of barrier function although exposed to a similar concentration of circulating mediators and blood vessel wall–derived signals? A likely explanation is different flow patterns.^157,158^ Interestingly, a recent report demonstrated that laminar shear stress triggers Dll4-dependent proteolytic activation of Notch1 to reveal the Notch1 transmembrane domain, the key domain that mediates barrier establishment.^159^ Expression of the Notch1 transmembrane domain is sufficient to rescue Notch1 knockout–induced defects in barrier function and does so by catalyzing the formation of a novel receptor complex in the plasma membrane containing VE-cadherin, a tight junction component.^159^ It seems, therefore, that physiological shear stress is a key stimulator for endothelial barrier function, as well as reduced cell turnover. Coinciding with this finding is the earlier report that in vitro laminar shear stress can directly stimulate stem cell differentiation toward ECs^160^ in the absence of VEGF, which would favor endothelial repair. In general, several key signaling pathways have been proposed for stem/progenitor cell commitment to endothelial lineage via VEGFR2 activation in a ligand-independent manner.^150,160,161^ How VEGFR2 can be activated by shear stress without VEGF involvement requires further investigation.

From a different perspective, Toledo-Flores et al^162^ observed that Sca-1^+^/CD45^+^ adventitial cells possess proangiogenic capacity and may be the source of the vasa vasorum expansion in atherosclerosis in mice. Although in vitro Sca-1^+^ cell culture can promote expression of endothelial markers, no data demonstrate endothelial lineage differentiation in vivo. However, Sca1^+^ progenitors can be forced to differentiate into ECs in vitro and in vivo via introduction of the gene *ETV2*.^163^ After ETV2 transduction, Sca-1^+^ adventitial cells significantly increased expression of early *EC* genes, including VE-cadherin, Flk1, and Tie2 at the mRNA and protein levels. ETV2 overexpression also induced the downregulation of a panel of SMC and mesenchymal genes through epigenetic regulations, mediated by decreased expression of DNA-modifying enzymes, 10-11 translocation dioxygenases.^163^ Adventitial Sca-1^+^ cells grafted on the adventitial side of wire-injured femoral arteries increased vascular wall hyperplasia compared with ungrafted control arteries. Arteries seeded with ETV2-transduced cells showed, on the contrary, reduced hyperplasia compared with controls.^163^ Genetic manipulation of vascular progenitors is, therefore, a promising approach to improve vascular function after endothelial injury.

### SMC Accumulation in Neointimal Lesions Derived From Stem Cells

#### Identification

**Figure 4.**
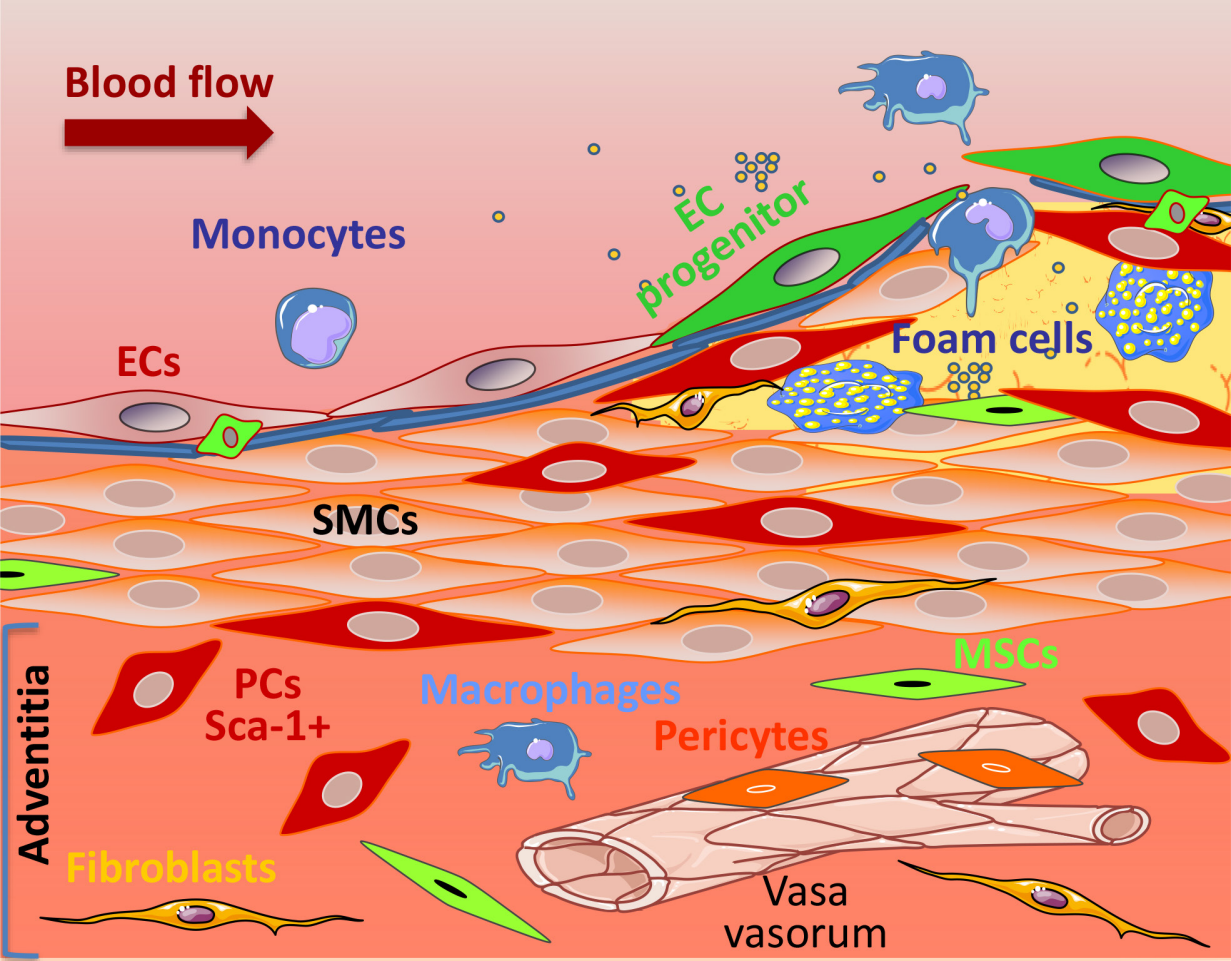
**Proposed roles for vascular wall resident progenitor cells (PCs) in lesion formation**. The adventitia is a dynamic layer in active communication with the other vessel wall layers, and it contains various cell types, including PCs (red), mesenchymal stem cells (MSCs in green), macrophages (blue), fibroblasts (yellow), and pericytes (orange) surrounding the adventitial vasa vasorum, among others. Adventitial PCs, expressing the stem cell marker Sca-1 (red), have the ability to migrate to the lesions and to differentiate into smooth muscle cells (SMCs), thus contributing to the intimal SMCs pool. EC indicates endothelial cell.

An important feature of the SMCs is their phenotypic plasticity, which enables them to respond to the changes of the surrounding environment. During vascular development or in response to injury, the medial SMCs adopt a synthetic, proliferative, and migratory phenotype that can contribute to intima formation.^164^ However, vascular stem/progenitor cells may also have roles in neointimal formation. For instance, Hu et al^165^ revealed that the origin of the SMCs in the neointimal lesion of vessel allografts was not bone marrow progenitor cells but locally derived host cells. These findings were further supported by the work on atherosclerosis. Bentzon et al^166,167^ concluded that there is a local vessel wall source of SMCs, and Iwata et al^168^ showed that bone marrow cells contribute to vascular inflammation but do not differentiate into SMCs. A role for adventitial Sca-1^+^ cells as progenitor cells for resident SMCs was supported by Passman et al,^19^ who characterized a sonic hedgehog signaling domain restricted to the adventitial layer of the vessel wall. In sonic hedgehog^−/−^ mice, the number of adventitial Sca-1^+^ cells was drastically reduced and, in parallel, the number of potential SMC progenitor cells was also decreased. Tsai et al^12^ performed a study aimed at exploring the contribution of stem/progenitor cells to neointima formation in decellularized vessel grafts in a mouse model. A decellularized vessel was grafted in the mouse carotid artery, and a neointimal lesion was formed. The lesion exhibited Sca-1^+^, c-Kit^+^, and CD34^+^ cells, with multilineage differentiation capacity.^12^ After femoral artery injury, a recent report concluded that >50% of neointimal SMCs are derived from adventitial stem/progenitor cells.^39^ Moreover, Roostalu et al^169^ used a double SMC-marker lineage tracing mouse model to provide solid evidence that adventitial cells are the main contributors to neointimal SMCs in severely injured arteries. Furthermore, genetic fate-tracing using Gli1 as a marker indicates that adventitial stem/progenitor-like cells migrate into the media and neointima during athero- and arteriosclerosis in ApoE^−/−^ mice. Hence, adventitial stem cells can be direct contributors to SMC accumulation in many forms of arteriosclerosis (Figure 4).

#### Cell Migration

**Figure 5.**
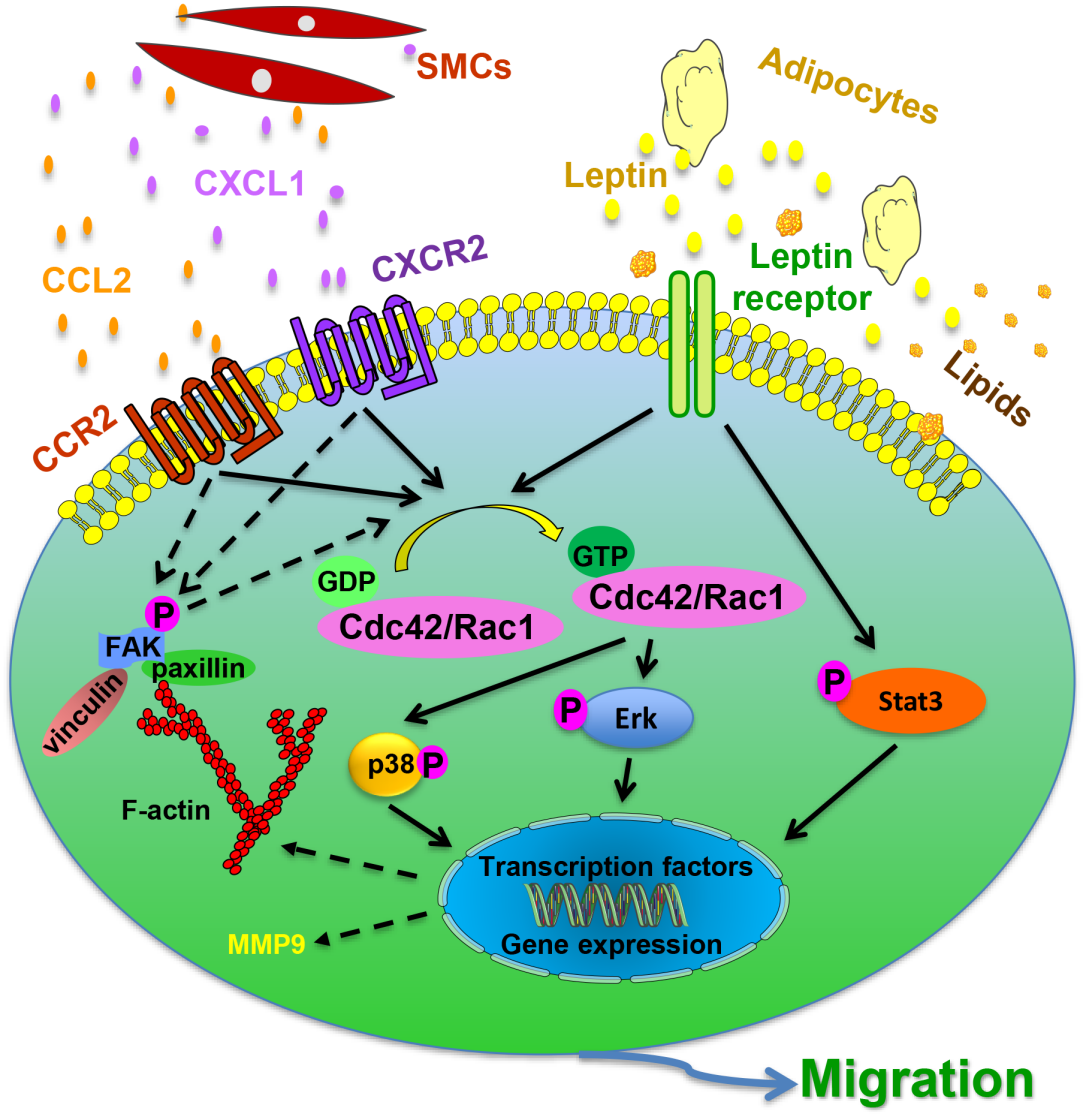
**Proposed mechanism of vascular wall resident Sca-1^+^ progenitor cell migration in response to chemokines and lipoproteins.** Sca-1^+^ vascular progenitor cells are resident in the vessel wall. Chemokines, such as CCL2 (chemokine [C-C motif] ligand) and CXCL1 (chemokine [C-X-C motif] ligand), are released by activated smooth muscle cells (SMCs) and act on the Sca-1^+^ cells by binding to their respective receptors, CCR2 (C-C chemokine receptor type) and CXCR2. As a result, downstream pathways are activated, which include GTPAses Rac1 and Cdc42, p38 is phosphorylated and cytoskeleton-related proteins upregulation (FAK [focal adhesion kinase], vinculin, paxillin). Adipocytes also release signals, such as leptin, which bind to the corresponding receptors expressed in Sca-1^+^ cells. Consequently, Rac1 and Cdc42 are activated, leading to ERK (extracellular signal–regulated kinase) phosphorylation. Leptin also activates Stat3 pathway and the expression of cytoskeleton-related proteins. Together, the activation of the mentioned signaling pathways leads to either transcription of migration-related genes or direct activation of the cytoskeleton to promote cell migration. Furthermore, lipid particles, such as low-density lipoprotein or cholesterol, may be incorporated by Sca-1^+^ cells and lead to increased migration by activating relevant migration pathways and inducing the expression of matrix degradation proteins by the expression of matrix metalloproteinase 9.

Perivascular application of GFP-Sca-1^+^ cells to injured arteries significantly enhanced neointimal lesion formation via progenitor migration.^121^ Application of Sca-1^+^ cells to the adventitial side of vessel grafts resulted in enhanced neointimal lesions.^29^ The potential of Sca-1^+^ cells to migrate from the adventitia to the intima was also demonstrated by the work of Wong et al^30^ using an ex vivo bioreactor system. Adventitial Sca-1^+^ cells were seeded on the outside of a decellularized vessel, and their migration toward the inner side of the vessel was increased in response to sirolimus stimulation.^30^ Another study by Chen et al^29^ showed that the adventitia of vein grafts, which underwent intense remodeling, displayed a considerable number of Sca-1^+^ progenitor cells near the vasa vasorum. These cells could differentiate in vitro into SMCs and also had the potential to differentiate into adipogenic, osteogenic, and chondrogenic lineages. Furthermore, ex vivo and in vivo experiments demonstrated migration of the Sca-1^+^ cells to the inner side of the vessels, making a significant contribution to the content of neointimal SMCs.^29^ The mechanisms driving stem/progenitor cell migration seem to involve several chemokines and lipoproteins, and great progress toward their understanding has been made recently. Using combined models, Yu et al^121^ demonstrated that proliferating SMCs can release several chemokines, including CXCL1 (chemokine [C-X-C motif] ligand), CCL2 (chemokine [C-C motif] ligand), and CCL5, which have a role in attracting vascular progenitors. Single cell tracking experiments indicated that these cells migrate directionally and efficiently but not randomly. Interestingly, Sca-1^+^ progenitor cell migration from the adventitia to the neointima was abrogated and neointima formation diminished, in a wire injury model using CCL2^−/−^ mice.^121^ These findings suggest that vascular stem/progenitor cell migration from the adventitia to the neointima can be induced by SMC release of chemokines, which act via CCR2 (C-C chemokine receptor type)/Rac1/p38 and CXCR2/Rac1/p38 signaling pathways (Figure 5).

#### Influence of Obesity

A recent publication reveals an effect of the adipokine, leptin, on Sca-1^+^ progenitor cell migration and neointimal formation.^155^ Leptin induced the migration of Sca-1^+^ progenitor cells in vitro, which was markedly reduced in cells derived from leptin receptor knockout mice. Moreover, transplantation of leptin receptor^+/+^ Sca-1^+^ progenitor cells into the adventitial side of injured artery in leptin receptor^−/−^ mice significantly enhanced neointimal formation. Upregulation of leptin levels in both the vessel wall and the circulation, after vessel injury, promoted the migration of Sca-1^+^ progenitor cells via leptin receptor–dependent STAT3-Rac1/Cdc42-ERK-FAK (focal adhesion kinase) pathways^155^ and enhanced neointimal formation (Figure 5).

#### Hyperlipidemia

Kokkinopoulos et al^120^ used single cell RNA sequencing technology on primary adventitial mouse Sca-1^+^ cells from wild-type and atherosclerotic-prone (ApoE^−/−^) mice and found that a group of genes controlling cell migration and matrix protein degradation was highly altered. Adventitial progenitors from ApoE^−/−^ mice displayed an augmented migratory potential both in vitro and in vivo. This increased migratory ability was mimicked by lipid loading either by incorporating oxidized low-density lipoprotein or cholesterol to Sca-1^+^ progenitor cells.^120^ Furthermore, lipid loading increased miRNA-29b expression and induced sirtuin-1 and MMP-9 (matrix metalloproteinase) levels to promote cell migration (Figure 5). These results provide mechanisms by which blood cholesterol levels could influence vascular progenitor cell migration, a promising target for the treatment of vascular diseases.

#### Differentiation Into SMCs

Concerning the mechanisms underlying SMC differentiation, recent studies reveal the involvement of collagen IV, integrins α1, α5, and β1, and PDGF-β receptor pathways.^170^ In addition, a novel finding is the proposed role of the cytokine-like protein, DKK3 (Dickkopf 3). DKK3 is a secreted glycoprotein, highly expressed in the endothelium and muscles,^171–174^ that is implicated in the differentiation of partially induced pluripotent stem cells.^175^ Recently, Cheng et al^176^ demonstrated that the loss of DKK3 attenuates atherosclerosis development in ApoE^−/−^ mice fed with high-fat diet through activation of the β-catenin signaling. However, Yu et al^177^ reported that DKK3 is an atheroprotective cytokine, based on a prospective human population study and on mouse models. Another study showed that the absence of DKK3 leads to vulnerable atherosclerotic plaques because of reduced numbers of SMCs and matrix protein deposition, as well as increased hemorrhage and macrophage infiltration.^123^ Furthermore, the in vitro studies revealed that DKK3 can induce the differentiation of Sca-1^+^ vascular progenitors into SMCs, via activation of the TGFβ/ATF6 (activating transcription factor) and Wnt signaling pathways. Wang et al^174^ also demonstrated that DKK3 can stimulate murine stem cell differentiation into SMCs by activating ATF6 and promoting myocardin expression. Cumulatively, DKK3 seems to be a potent SMC differentiation factor associated with plaque stability.^123^

#### Foam Cell Formation

Foam cells, a prominent component of advanced plaques, may be derived from SMCs or macrophages that have taken up lipids. Psaltis et al^41,42,178^ identified the presence of resident Sca-1^+^/CD45^+^ macrophage progenitor cells in the aortic adventitia. These progenitors were upregulated in ApoE^−/−^ and low-density lipoprotein^−/−^ hyperlipidemic mice and could form foam cells. As mentioned above, resident Sca-1^+^ progenitors can largely differentiate into SMCs, and these could also be a source of foam cells. Hence, local resident stem/progenitor cells could be a common progenitor for both SMCs and macrophage-derived foam cells.

## Summary and Perspectives

Accumulating evidence suggests the presence of stem/progenitor cells in the vessel wall. Several markers were used to identify these stem cells, for example, Sca-1, c-Kit, CD34, Flk1, Sox, CD124/90, among others, most of which have been listed in Table 2. However, there is no unique marker available to identify vascular stem/progenitor cells. It is also not clear whether all types of vascular stem cells are derived from the same source during development or whether they alter in adults, especially under disease conditions. To answer these questions, further investigations would be needed. Single cell gene sequencing and cell linear tracing models may be especially useful to clarify the nature and the contributions of vascular stem/progenitor cells during development and disease formation.

Recent reports indicate the presence of stem and progenitor cell populations within the endothelial and other layers of the vessel wall. Their definitions and functions should be investigated in detail in the future. For example, nonbone marrow–derived c-Kit^+^ cells may participate in angiogenesis in ischemic heart, as identified by cell lineage tracing models,^114–116^ but it is unknown whether vascular resident c-Kit^+^ cells have the ability to repair or regenerate the endothelium of large vessels. Because atherosclerotic plaques occur mainly in medium-to-large arteries, it will be crucial to study the role of vascular endothelial stem cells in the repair of damaged endothelium in these vessels.

**Figure 6.**
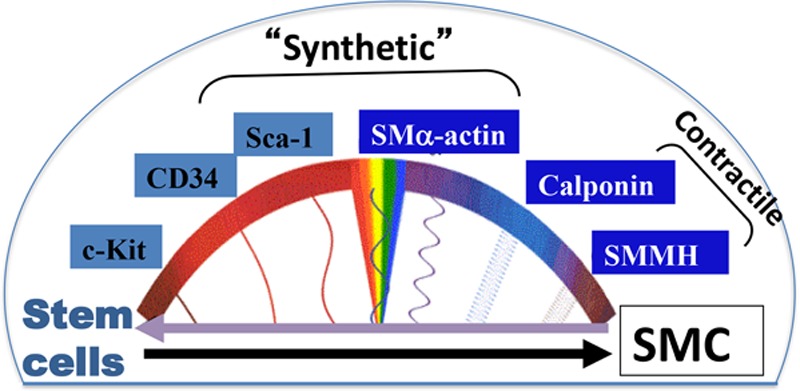
**Smooth muscle cell (SMC) heterogeneity and stem cell differentiation.** During vascular development or in response to injury, the media SMCs exhibit a synthetic, proliferative, and migratory phenotype, where they may express some stem cell markers (Sca-1) and also the marker SMα-actin. This is accompanied by a decrease in the expression of SMC differentiation markers, such as calponin and SMMHC (smooth muscle myosin heavy chain). Stem cells (Sca-1^+^, c-Kit^+^, CD34^+^) can differentiate into SMCs in response to vascular injury, and thus, their expression of stem cell markers is decreased, whereas the expression of SMC markers is increased (SMα-actin, calponin, SMMHC). Mature and fully differentiated SMCs express high levels of calponin and SMMHC and show reduced or negligent levels of stem cell markers. These cells present a more elongated morphology and a contractile phenotype, with a low proliferative and migratory rate. Stem/progenitor cell markers can potentially be expressed in dedifferentiated SMCs, which have gone through a dedifferentiation process in atherosclerosis, and which contribute to vessel wall remodeling.

It is well-known that SMCs within vascular lesions consist of heterogeneous populations, reflecting the variable phenotypes and functions of these cells.^179^ During development, SMCs derived from different regions, for example, neural crest cells versus proepicardium. How different origins of SMCs during the development affect their behavior during the formation of lesions in adults is a worthy question for future study. However, SMCs may be able to dedifferentiate or give rise to other cell types, including MSCs.^34,180^ A recent paper^181^ demonstrated that SMCs in injury-induced neointimal lesions and in atherosclerotic plaques were oligoclonal, having derived from a few expanding cells, which could be quiescent SMC progenitors or be able to dedifferentiate into progenitors. We could speculate that lesional SMCs comprise all the different stages of differentiation from stem cells, hence display a variety of phenotypes (Figure 6). However, further study is needed to answer what proportion of lesional SMCs is derived from stem cells. Further research is urgently needed to investigate the mechanisms driving the migration, proliferation, and differentiation of stem cells during vascular remodeling. The elucidation of these mechanisms should provide vital information needed for the development of more efficient or novel therapies for vascular diseases.

## Acknowledgments

We acknowledge Professor Andrew Newby for his valuable input on the revision of this manuscript. Figures 2–4 were prepared using Servier Medical Art image bank.

## Sources of Funding

This work was supported by British Heart Foundation (RG/14/6/31144), the Strategic Priority Research Program of the Chinese Academy of Sciences (CAS, XDA16020204, XDB19000000), Royal Society-Newton Advanced Fellowship (NA170109), and National Natural Science Foundation of China (81570249, 91639302, 91339102, and 91539103).

## Disclosures

None.

## References

[1] Flamme I, Frölich T, Risau W (1997). Molecular mechanisms of vasculogenesis and embryonic angiogenesis.. J Cell Physiol.

[2] Carmeliet P (2003). Angiogenesis in health and disease.. Nat Med.

[3] Kovacic JC, Moore J, Herbert A, Ma D, Boehm M, Graham RM (2008). Endothelial progenitor cells, angioblasts, and angiogenesis–old terms reconsidered from a current perspective.. Trends Cardiovasc Med.

[4] Ross R (1993). The pathogenesis of atherosclerosis: a perspective for the 1990s.. Nature.

[5] Libby P, Hansson GK (2015). Inflammation and immunity in diseases of the arterial tree: players and layers.. Circ Res.

[6] Alexander MR, Owens GK (2012). Epigenetic control of smooth muscle cell differentiation and phenotypic switching in vascular development and disease.. Annu Rev Physiol.

[7] Xu Q (2006). The impact of progenitor cells in atherosclerosis.. Nat Clin Pract Cardiovasc Med.

[8] Yu B, Chen Q, Le Bras A, Zhang L, Xu Q Vascular stem/progenitor cell migration and differentiation in atherosclerosis [published online ahead of print July 5, 2017].. Antioxid Redox Signal.

[9] Ingram DA, Mead LE, Moore DB, Woodard W, Fenoglio A, Yoder MC (2005). Vessel wall-derived endothelial cells rapidly proliferate because they contain a complete hierarchy of endothelial progenitor cells.. Blood.

[10] Torsney E, Mandal K, Halliday A, Jahangiri M, Xu Q (2007). Characterisation of progenitor cells in human atherosclerotic vessels.. Atherosclerosis.

[11] Naito H, Kidoya H, Sakimoto S, Wakabayashi T, Takakura N (2012). Identification and characterization of a resident vascular stem/progenitor cell population in preexisting blood vessels.. EMBO J.

[12] Tsai TN, Kirton JP, Campagnolo P, Zhang L, Xiao Q, Zhang Z, Wang W, Hu Y, Xu Q (2012). Contribution of stem cells to neointimal formation of decellularized vessel grafts in a novel mouse model.. Am J Pathol.

[13] Fang S, Wei J, Pentinmikko N, Leinonen H, Salven P (2012). Generation of functional blood vessels from a single c-kit+ adult vascular endothelial stem cell.. PLoS Biol.

[14] Patel J, Seppanen EJ, Rodero MP, Wong HY, Donovan P, Neufeld Z, Fisk NM, Francois M, Khosrotehrani K (2017). Functional definition of progenitors versus mature endothelial cells reveals key SoxF-dependent differentiation process.. Circulation.

[15] Green L, Ofstein RH, Rapp B, Saadatzadeh MR, Bhavsar JR, Fajardo A, Dalsing MC, Ingram DA, Murphy MP (2017). Adult venous endothelium is a niche for highly proliferative and vasculogenic endothelial colony-forming cells.. J Vasc Surg.

[16] Sainz J, Al Haj Zen A, Caligiuri G, Demerens C, Urbain D, Lemitre M, Lafont A (2006). Isolation of “side population” progenitor cells from healthy arteries of adult mice.. Arterioscler Thromb Vasc Biol.

[17] Zengin E, Chalajour F, Gehling UM, Ito WD, Treede H, Lauke H, Weil J, Reichenspurner H, Kilic N, Ergün S (2006). Vascular wall resident progenitor cells: a source for postnatal vasculogenesis.. Development.

[18] Pasquinelli G, Tazzari PL, Vaselli C, Foroni L, Buzzi M, Storci G, Alviano F, Ricci F, Bonafè M, Orrico C, Bagnara GP, Stella A, Conte R (2007). Thoracic aortas from multiorgan donors are suitable for obtaining resident angiogenic mesenchymal stromal cells.. Stem Cells.

[19] Passman JN, Dong XR, Wu SP, Maguire CT, Hogan KA, Bautch VL, Majesky MW (2008). A sonic hedgehog signaling domain in the arterial adventitia supports resident Sca1+ smooth muscle progenitor cells.. Proc Natl Acad Sci USA.

[20] Tang Z, Wang A, Yuan F, Yan Z, Liu B, Chu JS, Helms JA, Li S (2012). Differentiation of multipotent vascular stem cells contributes to vascular diseases.. Nat Commun.

[21] Pasquinelli G, Pacilli A, Alviano F, Foroni L, Ricci F, Valente S, Orrico C, Lanzoni G, Buzzi M, Luigi Tazzari P, Pagliaro P, Stella A, Paolo Bagnara G (2010). Multidistrict human mesenchymal vascular cells: pluripotency and stemness characteristics.. Cytotherapy.

[22] Zaniboni A, Bernardini C, Alessandri M, Mangano C, Zannoni A, Bianchi F, Sarli G, Calzà L, Bacci ML, Forni M (2014). Cells derived from porcine aorta tunica media show mesenchymal stromal-like cell properties in in vitro culture.. Am J Physiol Cell Physiol.

[23] Evans JF, Salvador V, George S, Trevino-Gutierrez C, Nunez C (2015). Mouse aorta-derived mesenchymal progenitor cells contribute to and enhance the immune response of macrophage cells under inflammatory conditions.. Stem Cell Res Ther.

[24] Zorzi P, Aplin AC, Smith KD, Nicosia RF (2010). Technical advance: the rat aorta contains resident mononuclear phagocytes with proliferative capacity and proangiogenic properties.. J Leukoc Biol.

[25] Sawada H, Rateri DL, Moorleghen JJ, Majesky MW, Daugherty A (2017). Smooth muscle cells derived from second heart field and cardiac neural crest reside in spatially distinct domains in the media of the ascending aorta-brief report.. Arterioscler Thromb Vasc Biol.

[26] Hu Y, Zhang Z, Torsney E, Afzal AR, Davison F, Metzler B, Xu Q (2004). Abundant progenitor cells in the adventitia contribute to atherosclerosis of vein grafts in ApoE-deficient mice.. J Clin Invest.

[27] Klein D, Weisshardt P, Kleff V, Jastrow H, Jakob HG, Ergün S (2011). Vascular wall-resident CD44+ multipotent stem cells give rise to pericytes and smooth muscle cells and contribute to new vessel maturation.. PLoS One.

[28] Klein D, Benchellal M, Kleff V, Jakob HG, Ergün S (2013). Hox genes are involved in vascular wall-resident multipotent stem cell differentiation into smooth muscle cells.. Sci Rep.

[29] Chen Y, Wong MM, Campagnolo P, Simpson R, Winkler B, Margariti A, Hu Y, Xu Q (2013). Adventitial stem cells in vein grafts display multilineage potential that contributes to neointimal formation.. Arterioscler Thromb Vasc Biol.

[30] Wong MM, Winkler B, Karamariti E, Wang X, Yu B, Simpson R, Chen T, Margariti A, Xu Q (2013). Sirolimus stimulates vascular stem/progenitor cell migration and differentiation into smooth muscle cells via epidermal growth factor receptor/extracellular signal-regulated kinase/β-catenin signaling pathway.. Arterioscler Thromb Vasc Biol.

[31] Klein D, Meissner N, Kleff V, Jastrow H, Yamaguchi M, Ergün S, Jendrossek V (2014). Nestin(+) tissue-resident multipotent stem cells contribute to tumor progression by differentiating into pericytes and smooth muscle cells resulting in blood vessel remodeling.. Front Oncol.

[32] Wu Y, Shen Y, Kang K, Zhang Y, Ao F, Wan Y, Song J (2015). Effects of estrogen on growth and smooth muscle differentiation of vascular wall-resident CD34(+) stem/progenitor cells.. Atherosclerosis.

[33] Shen Y, Wu Y, Zheng Y, Ao F, Kang K, Wan Y, Song J (2016). Responses of adventitial CD34+ vascular wall-resident stem/progenitor cells and medial smooth muscle cells to carotid injury in rats.. Exp Mol Pathol.

[34] Majesky MW, Horita H, Ostriker A, Lu S, Regan JN, Bagchi A, Dong XR, Poczobutt J, Nemenoff RA, Weiser-Evans MC (2017). Differentiated smooth muscle cells generate a subpopulation of resident vascular progenitor cells in the adventitia regulated by Klf4.. Circ Res.

[35] Hoshino A, Chiba H, Nagai K, Ishii G, Ochiai A (2008). Human vascular adventitial fibroblasts contain mesenchymal stem/progenitor cells.. Biochem Biophys Res Commun.

[36] Campagnolo P, Cesselli D, Al Haj Zen A, Beltrami AP, Kränkel N, Katare R, Angelini G, Emanueli C, Madeddu P (2010). Human adult vena saphena contains perivascular progenitor cells endowed with clonogenic and proangiogenic potential.. Circulation.

[37] Corselli M, Chen CW, Sun B, Yap S, Rubin JP, Péault B (2012). The tunica adventitia of human arteries and veins as a source of mesenchymal stem cells.. Stem Cells Dev.

[38] Tigges U, Komatsu M, Stallcup WB (2013). Adventitial pericyte progenitor/mesenchymal stem cells participate in the restenotic response to arterial injury.. J Vasc Res.

[39] Kramann R, Goettsch C, Wongboonsin J, Iwata H, Schneider RK, Kuppe C, Kaesler N, Chang-Panesso M, Machado FG, Gratwohl S, Madhurima K, Hutcheson JD, Jain S, Aikawa E, Humphreys BD (2016). Adventitial MSC-like cells are progenitors of vascular smooth muscle cells and drive vascular calcification in chronic kidney disease.. Cell Stem Cell.

[40] Kramann R, Schneider RK, DiRocco DP, Machado F, Fleig S, Bondzie PA, Henderson JM, Ebert BL, Humphreys BD (2015). Perivascular Gli1+ progenitors are key contributors to injury-induced organ fibrosis.. Cell Stem Cell.

[41] Psaltis PJ, Harbuzariu A, Delacroix S, Witt TA, Holroyd EW, Spoon DB, Hoffman SJ, Pan S, Kleppe LS, Mueske CS, Gulati R, Sandhu GS, Simari RD (2012). Identification of a monocyte-predisposed hierarchy of hematopoietic progenitor cells in the adventitia of postnatal murine aorta.. Circulation.

[42] Psaltis PJ, Puranik AS, Spoon DB, Chue CD, Hoffman SJ, Witt TA, Delacroix S, Kleppe LS, Mueske CS, Pan S, Gulati R, Simari RD (2014). Characterization of a resident population of adventitial macrophage progenitor cells in postnatal vasculature.. Circ Res.

[43] Wörsdörfer P, Mekala SR, Bauer J, Edenhofer F, Kuerten S, Ergün S (2017). The vascular adventitia: an endogenous, omnipresent source of stem cells in the body.. Pharmacol Ther.

[44] Xu Q (2008). Stem cells and transplant arteriosclerosis.. Circ Res.

[45] Carmeliet P (2005). Angiogenesis in life, disease and medicine.. Nature.

[46] Ding BS, Nolan DJ, Butler JM, James D, Babazadeh AO, Rosenwaks Z, Mittal V, Kobayashi H, Shido K, Lyden D, Sato TN, Rabbany SY, Rafii S (2010). Inductive angiocrine signals from sinusoidal endothelium are required for liver regeneration.. Nature.

[47] Sabin F (1917). Preliminary note on the differentiation of angioblasts and the method by which they produce blood-vessels, blood-plasma and red blood-cells as seen in the living chick.. Anat Rec.

[48] Risau W (1997). Mechanisms of angiogenesis.. Nature.

[49] Ema M, Takahashi S, Rossant J (2006). Deletion of the selection cassette, but not cis-acting elements, in targeted Flk1-lacZ allele reveals Flk1 expression in multipotent mesodermal progenitors.. Blood.

[50] Minasi MG, Riminucci M, De Angelis L, Borello U, Berarducci B, Innocenzi A, Caprioli A, Sirabella D, Baiocchi M, De Maria R, Boratto R, Jaffredo T, Broccoli V, Bianco P, Cossu G (2002). The meso-angioblast: a multipotent, self-renewing cell that originates from the dorsal aorta and differentiates into most mesodermal tissues.. Development.

[51] Condorelli G, Borello U, De Angelis L (2001). Cardiomyocytes induce endothelial cells to trans-differentiate into cardiac muscle: implications for myocardium regeneration.. Proc Natl Acad Sci USA.

[52] Kardon G, Campbell JK, Tabin CJ (2002). Local extrinsic signals determine muscle and endothelial cell fate and patterning in the vertebrate limb.. Dev Cell.

[53] Esner M, Meilhac SM, Relaix F, Nicolas JF, Cossu G, Buckingham ME (2006). Smooth muscle of the dorsal aorta shares a common clonal origin with skeletal muscle of the myotome.. Development.

[54] Wu SM, Fujiwara Y, Cibulsky SM, Clapham DE, Lien CL, Schultheiss TM, Orkin SH (2006). Developmental origin of a bipotential myocardial and smooth muscle cell precursor in the mammalian heart.. Cell.

[55] Motoike T, Markham DW, Rossant J, Sato TN (2003). Evidence for novel fate of Flk1+ progenitor: contribution to muscle lineage.. Genesis.

[56] Sampaolesi M, Torrente Y, Innocenzi A, Tonlorenzi R, D’Antona G, Pellegrino MA, Barresi R, Bresolin N, De Angelis MG, Campbell KP, Bottinelli R, Cossu G (2003). Cell therapy of alpha-sarcoglycan null dystrophic mice through intra-arterial delivery of mesoangioblasts.. Science.

[57] Cossu G, Bianco P (2003). Mesoangioblasts–vascular progenitors for extravascular mesodermal tissues.. Curr Opin Genet Dev.

[58] Choi K, Kennedy M, Kazarov A, Papadimitriou JC, Keller G (1998). A common precursor for hematopoietic and endothelial cells.. Development.

[59] Huber TL, Kouskoff V, Fehling HJ, Palis J, Keller G (2004). Haemangioblast commitment is initiated in the primitive streak of the mouse embryo.. Nature.

[60] Vogeli KM, Jin SW, Martin GR, Stainier DY (2006). A common progenitor for haematopoietic and endothelial lineages in the zebrafish gastrula.. Nature.

[61] Kinder SJ, Tsang TE, Quinlan GA, Hadjantonakis AK, Nagy A, Tam PP (1999). The orderly allocation of mesodermal cells to the extraembryonic structures and the anteroposterior axis during gastrulation of the mouse embryo.. Development.

[62] Lacaud G, Kouskoff V (2017). Hemangioblast, hemogenic endothelium, and primitive versus definitive hematopoiesis.. Exp Hematol.

[63] Ueno H, Weissman IL (2006). Clonal analysis of mouse development reveals a polyclonal origin for yolk sac blood islands.. Dev Cell.

[64] Shalaby F, Rossant J, Yamaguchi TP, Gertsenstein M, Wu XF, Breitman ML, Schuh AC (1995). Failure of blood-island formation and vasculogenesis in Flk-1-deficient mice.. Nature.

[65] Fong GH, Rossant J, Gertsenstein M, Breitman ML (1995). Role of the Flt-1 receptor tyrosine kinase in regulating the assembly of vascular endothelium.. Nature.

[66] Shalaby F, Ho J, Stanford WL, Fischer KD, Schuh AC, Schwartz L, Bernstein A, Rossant J (1997). A requirement for Flk1 in primitive and definitive hematopoiesis and vasculogenesis.. Cell.

[67] Armulik A, Genové G, Betsholtz C (2011). Pericytes: developmental, physiological, and pathological perspectives, problems, and promises.. Dev Cell.

[68] Cai CL, Liang X, Shi Y, Chu PH, Pfaff SL, Chen J, Evans S (2003). Isl1 identifies a cardiac progenitor population that proliferates prior to differentiation and contributes a majority of cells to the heart.. Dev Cell.

[69] Sun Y, Liang X, Najafi N, Cass M, Lin L, Cai CL, Chen J, Evans SM (2007). Islet 1 is expressed in distinct cardiovascular lineages, including pacemaker and coronary vascular cells.. Dev Biol.

[70] Zhou B, Pu WT (2008). More than a cover: epicardium as a novel source of cardiac progenitor cells.. Regen Med.

[71] Wasteson P, Johansson BR, Jukkola T, Breuer S, Akyürek LM, Partanen J, Lindahl P (2008). Developmental origin of smooth muscle cells in the descending aorta in mice.. Development.

[72] Chen D, Xia Y, Zuo K, Wang Y, Zhang S, Kuang D, Duan Y, Zhao X, Wang G (2015). Crosstalk between SDF-1/CXCR4 and SDF-1/CXCR7 in cardiac stem cell migration.. Sci Rep.

[73] Dalton ML, Gadson PF, Wrenn RW, Rosenquist TH (1997). Homocysteine signal cascade: production of phospholipids, activation of protein kinase C, and the induction of c-fos and c-myb in smooth muscle cells.. FASEB J.

[74] Leroux-Berger M, Queguiner I, Maciel TT, Ho A, Relaix F, Kempf H (2011). Pathologic calcification of adult vascular smooth muscle cells differs on their crest or mesodermal embryonic origin.. J Bone Miner Res.

[75] Cheung C, Bernardo AS, Trotter MW, Pedersen RA, Sinha S (2012). Generation of human vascular smooth muscle subtypes provides insight into embryological origin-dependent disease susceptibility.. Nat Biotechnol.

[76] Wilm B, Ipenberg A, Hastie ND, Burch JB, Bader DM (2005). The serosal mesothelium is a major source of smooth muscle cells of the gut vasculature.. Development.

[77] Gittenberger-de Groot AC, Vrancken Peeters MP, Mentink MM, Gourdie RG, Poelmann RE (1998). Epicardium-derived cells contribute a novel population to the myocardial wall and the atrioventricular cushions.. Circ Res.

[78] Tian X, Pu WT, Zhou B (2015). Cellular origin and developmental program of coronary angiogenesis.. Circ Res.

[79] Moore-Morris T, Guimarães-Camboa N, Banerjee I (2014). Resident fibroblast lineages mediate pressure overload-induced cardiac fibrosis.. J Clin Invest.

[80] Compton LA, Potash DA, Brown CB, Barnett JV (2007). Coronary vessel development is dependent on the type III transforming growth factor beta receptor.. Circ Res.

[81] del Monte G, Casanova JC, Guadix JA, MacGrogan D, Burch JB, Pérez-Pomares JM, de la Pompa JL (2011). Differential Notch signaling in the epicardium is required for cardiac inflow development and coronary vessel morphogenesis.. Circ Res.

[82] Mellgren AM, Smith CL, Olsen GS, Eskiocak B, Zhou B, Kazi MN, Ruiz FR, Pu WT, Tallquist MD (2008). Platelet-derived growth factor receptor beta signaling is required for efficient epicardial cell migration and development of two distinct coronary vascular smooth muscle cell populations.. Circ Res.

[83] Lavine KJ, White AC, Park C, Smith CS, Choi K, Long F, Hui CC, Ornitz DM (2006). Fibroblast growth factor signals regulate a wave of Hedgehog activation that is essential for coronary vascular development.. Genes Dev.

[84] Volz KS, Jacobs AH, Chen HI, Poduri A, McKay AS, Riordan DP, Kofler N, Kitajewski J, Weissman I, Red-Horse K (2015). Pericytes are progenitors for coronary artery smooth muscle.. Elife.

[85] Chen Q, Zhang H, Liu Y, Adams S, Eilken H, Stehling M, Corada M, Dejana E, Zhou B, Adams RH (2016). Endothelial cells are progenitors of cardiac pericytes and vascular smooth muscle cells.. Nat Commun.

[86] Zhou B, Honor LB, He H (2011). Adult mouse epicardium modulates myocardial injury by secreting paracrine factors.. J Clin Invest.

[87] Yamashita J, Itoh H, Hirashima M, Ogawa M, Nishikawa S, Yurugi T, Naito M, Nakao K (2000). Flk1-positive cells derived from embryonic stem cells serve as vascular progenitors.. Nature.

[88] Moretti A, Caron L, Nakano A, Lam JT, Bernshausen A, Chen Y, Qyang Y, Bu L, Sasaki M, Martin-Puig S, Sun Y, Evans SM, Laugwitz KL, Chien KR (2006). Multipotent embryonic isl1+ progenitor cells lead to cardiac, smooth muscle, and endothelial cell diversification.. Cell.

[89] Kattman SJ, Huber TL, Keller GM (2006). Multipotent flk-1+ cardiovascular progenitor cells give rise to the cardiomyocyte, endothelial, and vascular smooth muscle lineages.. Dev Cell.

[90] Cano E, Carmona R, Ruiz-Villalba A, Rojas A, Chau YY, Wagner KD, Wagner N, Hastie ND, Muñoz-Chápuli R, Pérez-Pomares JM (2016). Extracardiac septum transversum/proepicardial endothelial cells pattern embryonic coronary arterio-venous connections.. Proc Natl Acad Sci USA.

[91] Yu QC, Song W, Wang D, Zeng YA (2016). Identification of blood vascular endothelial stem cells by the expression of protein C receptor.. Cell Res.

[92] Alessandri G, Girelli M, Taccagni G, Colombo A, Nicosia R, Caruso A, Baronio M, Pagano S, Cova L, Parati E (2001). Human vasculogenesis ex vivo: embryonal aorta as a tool for isolation of endothelial cell progenitors.. Lab Invest.

[93] Majka SM, Jackson KA, Kienstra KA, Majesky MW, Goodell MA, Hirschi KK (2003). Distinct progenitor populations in skeletal muscle are bone marrow derived and exhibit different cell fates during vascular regeneration.. J Clin Invest.

[94] Tintut Y, Alfonso Z, Saini T, Radcliff K, Watson K, Boström K, Demer LL (2003). Multilineage potential of cells from the artery wall.. Circulation.

[95] Invernici G, Emanueli C, Madeddu P (2007). Human fetal aorta contains vascular progenitor cells capable of inducing vasculogenesis, angiogenesis, and myogenesis in vitro and in a murine model of peripheral ischemia.. Am J Pathol.

[96] Traktuev DO, Merfeld-Clauss S, Li J, Kolonin M, Arap W, Pasqualini R, Johnstone BH, March KL (2008). A population of multipotent CD34-positive adipose stromal cells share pericyte and mesenchymal surface markers, reside in a periendothelial location, and stabilize endothelial networks.. Circ Res.

[97] Crisan M, Yap S, Casteilla L (2008). A perivascular origin for mesenchymal stem cells in multiple human organs.. Cell Stem Cell.

[98] Barcelos LS, Duplaa C, Kränkel N (2009). Human CD133+ progenitor cells promote the healing of diabetic ischemic ulcers by paracrine stimulation of angiogenesis and activation of Wnt signaling.. Circ Res.

[99] Bearzi C, Leri A, Lo Monaco F (2009). Identification of a coronary vascular progenitor cell in the human heart.. Proc Natl Acad Sci USA.

[100] Juchem G, Weiss DR, Gansera B, Kemkes BM, Mueller-Hoecker J, Nees S (2010). Pericytes in the macrovascular intima: possible physiological and pathogenetic impact.. Am J Physiol Heart Circ Physiol.

[101] Katare R, Riu F, Mitchell K, Gubernator M, Campagnolo P, Cui Y, Fortunato O, Avolio E, Cesselli D, Beltrami AP, Angelini G, Emanueli C, Madeddu P (2011). Transplantation of human pericyte progenitor cells improves the repair of infarcted heart through activation of an angiogenic program involving micro-RNA-132.. Circ Res.

[102] Montiel-Eulefi E, Nery AA, Rodrigues LC, Sánchez R, Romero F, Ulrich H (2012). Neural differentiation of rat aorta pericyte cells.. Cytometry A.

[103] Yang S, Eto H, Kato H, Doi K, Kuno S, Kinoshita K, Ma H, Tsai CH, Chou WT, Yoshimura K (2013). Comparative characterization of stromal vascular cells derived from three types of vascular wall and adipose tissue.. Tissue Eng Part A.

[104] Iacobazzi D, Mangialardi G, Gubernator M, Hofner M, Wielscher M, Vierlinger K, Reni C, Oikawa A, Spinetti G, Vono R, Sangalli E, Montagnani M, Madeddu P (2014). Increased antioxidant defense mechanism in human adventitia-derived progenitor cells is associated with therapeutic benefit in ischemia.. Antioxid Redox Signal.

[105] Prandi F, Piola M, Soncini M, Colussi C, D’Alessandra Y, Penza E, Agrifoglio M, Vinci MC, Polvani G, Gaetano C, Fiore GB, Pesce M (2015). Adventitial vessel growth and progenitor cells activation in an ex vivo culture system mimicking human saphenous vein wall strain after coronary artery bypass grafting.. PLoS One.

[106] Sitnik KM, Wendland K, Weishaupt H, Uronen-Hansson H, White AJ, Anderson G, Kotarsky K, Agace WW (2016). Context-dependent development of lymphoid stroma from adult CD34(+) adventitial progenitors.. Cell Rep.

[107] Medina RJ, Barber CL, Sabatier F, Dignat-George F, Melero-Martin JM, Khosrotehrani K, Ohneda O, Randi AM, Chan JKY, Yamaguchi T, Van Hinsbergh VWM, Yoder MC, Stitt AW (2017). Endothelial progenitors: a consensus statement on nomenclature.. Stem Cells Transl Med.

[108] Covas DT, Piccinato CE, Orellana MD, Siufi JL, Silva WA, Proto-Siqueira R, Rizzatti EG, Neder L, Silva AR, Rocha V, Zago MA (2005). Mesenchymal stem cells can be obtained from the human saphena vein.. Exp Cell Res.

[109] Howson KM, Aplin AC, Gelati M, Alessandri G, Parati EA, Nicosia RF (2005). The postnatal rat aorta contains pericyte progenitor cells that form spheroidal colonies in suspension culture.. Am J Physiol Cell Physiol.

[110] Chen CW, Okada M, Proto JD, Gao X, Sekiya N, Beckman SA, Corselli M, Crisan M, Saparov A, Tobita K, Péault B, Huard J (2013). Human pericytes for ischemic heart repair.. Stem Cells.

[111] Visvader JE, Clevers H (2016). Tissue-specific designs of stem cell hierarchies.. Nat Cell Biol.

[112] Yoder MC (2010). Is endothelium the origin of endothelial progenitor cells?. Arterioscler Thromb Vasc Biol.

[113] Yoder MC (2018). Endothelial stem and progenitor cells (stem cells): (2017 Grover Conference Series).. Pulm Circ.

[114] van Berlo JH, Kanisicak O, Maillet M, Vagnozzi RJ, Karch J, Lin SC, Middleton RC, Marbán E, Molkentin JD (2014). c-kit+ cells minimally contribute cardiomyocytes to the heart.. Nature.

[115] Sultana N, Zhang L, Yan J, Chen J, Cai W, Razzaque S, Jeong D, Sheng W, Bu L, Xu M, Huang GY, Hajjar RJ, Zhou B, Moon A, Cai CL (2015). Resident c-kit(+) cells in the heart are not cardiac stem cells.. Nat Commun.

[116] Liu Q, Yang R, Huang X, Zhang H, He L, Zhang L, Tian X, Nie Y, Hu S, Yan Y, Zhang L, Qiao Z, Wang QD, Lui KO, Zhou B (2016). Genetic lineage tracing identifies in situ Kit-expressing cardiomyocytes.. Cell Res.

[117] Ingram DA, Caplice NM, Yoder MC (2005). Unresolved questions, changing definitions, and novel paradigms for defining endothelial progenitor cells.. Blood.

[118] Ma X, Robin C, Ottersbach K, Dzierzak E (2002). The Ly-6A (Sca-1) GFP transgene is expressed in all adult mouse hematopoietic stem cells.. Stem Cells.

[119] Foteinos G, Hu Y, Xiao Q, Metzler B, Xu Q (2008). Rapid endothelial turnover in atherosclerosis-prone areas coincides with stem cell repair in apolipoprotein E-deficient mice.. Circulation.

[120] Kokkinopoulos I, Wong MM, Potter CMF, Xie Y, Yu B, Warren DT, Nowak WN, Le Bras A, Ni Z, Zhou C, Ruan X, Karamariti E, Hu Y, Zhang L, Xu Q (2017). Adventitial SCA-1+ progenitor cell gene sequencing reveals the mechanisms of cell migration in response to hyperlipidemia.. Stem Cell Reports.

[121] Yu B, Wong MM, Potter CM, Simpson RM, Karamariti E, Zhang Z, Zeng L, Warren D, Hu Y, Wang W, Xu Q (2016). Vascular stem/progenitor cell migration induced by smooth muscle cell-derived chemokine (C-C Motif) ligand 2 and chemokine (C-X-C motif) ligand 1 contributes to neointima formation.. Stem Cells.

[122] Wong MM, Chen Y, Margariti A, Winkler B, Campagnolo P, Potter C, Hu Y, Xu Q (2014). Macrophages control vascular stem/progenitor cell plasticity through tumor necrosis factor-α-mediated nuclear factor-κB activation.. Arterioscler Thromb Vasc Biol.

[123] Karamariti E, Zhai C, Yu B (2018). DKK3 (Dickkopf 3) alters atherosclerotic plaque phenotype involving vascular progenitor and fibroblast differentiation into smooth muscle cells.. Arterioscler Thromb Vasc Biol.

[124] Campagnolo P, Tsai TN, Hong X, Kirton JP, So PW, Margariti A, Di Bernardini E, Wong MM, Hu Y, Stevens MM, Xu Q (2015). c-Kit+ progenitors generate vascular cells for tissue-engineered grafts through modulation of the Wnt/Klf4 pathway.. Biomaterials.

[125] Abedin M, Tintut Y, Demer LL (2004). Mesenchymal stem cells and the artery wall.. Circ Res.

[126] Ding DC, Shyu WC, Lin SZ (2011). Mesenchymal stem cells.. Cell Transplant.

[127] Pittenger MF, Mackay AM, Beck SC, Jaiswal RK, Douglas R, Mosca JD, Moorman MA, Simonetti DW, Craig S, Marshak DR (1999). Multilineage potential of adult human mesenchymal stem cells.. Science.

[128] Preda MB, Rønningen T, Burlacu A, Simionescu M, Moskaug JØ, Valen G (2014). Remote transplantation of mesenchymal stem cells protects the heart against ischemia-reperfusion injury.. Stem Cells.

[129] Planat-Benard V, Silvestre JS, Cousin B, André M, Nibbelink M, Tamarat R, Clergue M, Manneville C, Saillan-Barreau C, Duriez M, Tedgui A, Levy B, Pénicaud L, Casteilla L (2004). Plasticity of human adipose lineage cells toward endothelial cells: physiological and therapeutic perspectives.. Circulation.

[130] Tang W, Zeve D, Suh JM, Bosnakovski D, Kyba M, Hammer RE, Tallquist MD, Graff JM (2008). White fat progenitor cells reside in the adipose vasculature.. Science.

[131] Yoshimura K, Shigeura T, Matsumoto D, Sato T, Takaki Y, Aiba-Kojima E, Sato K, Inoue K, Nagase T, Koshima I, Gonda K (2006). Characterization of freshly isolated and cultured cells derived from the fatty and fluid portions of liposuction aspirates.. J Cell Physiol.

[132] Bailey AM, Kapur S, J Katz A (2010). Characterization of adipose-derived stem cells: an update.. Curr Stem Cell Res Ther.

[133] Olea FD, Locatelli P, Hnatiuk A, De Lorenzi A, Valdivieso L, Rocha E, Ramírez R, Laguens R, Crottogini A (2015). Vascular endothelial growth factor overexpression does not enhance adipose stromal cell-induced protection on muscle damage in critical limb ischemia.. Arterioscler Thromb Vasc Biol.

[134] Joe AW, Yi L, Even Y, Vogl AW, Rossi FM (2009). Depot-specific differences in adipogenic progenitor abundance and proliferative response to high-fat diet.. Stem Cells.

[135] Miranville A, Heeschen C, Sengenès C, Curat CA, Busse R, Bouloumié A (2004). Improvement of postnatal neovascularization by human adipose tissue-derived stem cells.. Circulation.

[136] Crisan M, Corselli M, Chen WC, Péault B (2012). Perivascular cells for regenerative medicine.. J Cell Mol Med.

[137] Krautler NJ, Kana V, Kranich J (2012). Follicular dendritic cells emerge from ubiquitous perivascular precursors.. Cell.

[138] Dellavalle A, Sampaolesi M, Tonlorenzi R (2007). Pericytes of human skeletal muscle are myogenic precursors distinct from satellite cells.. Nat Cell Biol.

[139] Guimaraes-Camboa N, Cattaneo P, Sun Y, Moore-Morris T, Gu Y, Dalton ND, Rockenstein E, Masliah E, Peterson KL, Stallcup WB, Chen J, Evans SM (2017). Pericytes of multiple organs do not behave as mesenchymal stem cells in vivo.. Cell Stem Cell.

[140] Cano E, Gebala V, Gerhardt H (2017). Pericytes or mesenchymal stem cells: is that the question?. Cell Stem Cell.

[141] Guimarães-Camboa N, Evans SM (2017). Are perivascular adipocyte progenitors mural cells or adventitial fibroblasts?. Cell Stem Cell.

[142] Stary HC, Chandler AB, Dinsmore RE, Fuster V, Glagov S, Insull W, Rosenfeld ME, Schwartz CJ, Wagner WD, Wissler RW (1995). A definition of advanced types of atherosclerotic lesions and a histological classification of atherosclerosis. A report from the Committee on Vascular Lesions of the Council on Arteriosclerosis, American Heart Association.. Arterioscler Thromb Vasc Biol.

[143] Stary HC, Chandler AB, Glagov S, Guyton JR, Insull W, Rosenfeld ME, Schaffer SA, Schwartz CJ, Wagner WD, Wissler RW (1994). A definition of initial, fatty streak, and intermediate lesions of atherosclerosis. A report from the Committee on Vascular Lesions of the Council on Arteriosclerosis, American Heart Association.. Circulation.

[144] Ross R (1999). Atherosclerosis–an inflammatory disease.. N Engl J Med.

[145] Talukder MA, Johnson WM, Varadharaj S, Lian J, Kearns PN, El-Mahdy MA, Liu X, Zweier JL (2011). Chronic cigarette smoking causes hypertension, increased oxidative stress, impaired NO bioavailability, endothelial dysfunction, and cardiac remodeling in mice.. Am J Physiol Heart Circ Physiol.

[146] Xiao Q, Luo Z, Pepe AE, Margariti A, Zeng L, Xu Q (2009). Embryonic stem cell differentiation into smooth muscle cells is mediated by Nox4-produced H2O2.. Am J Physiol Cell Physiol.

[147] Burtenshaw D, Hakimjavadi R, Redmond EM, Cahill PA (2017). Nox, reactive oxygen species and regulation of vascular cell fate.. Antioxidants.

[148] Zhou Y, Yan H, Guo M, Zhu J, Xiao Q, Zhang L (2013). Reactive oxygen species in vascular formation and development.. Oxid Med Cell Longev.

[149] Xiao Q, Zeng L, Zhang Z, Margariti A, Ali ZA, Channon KM, Xu Q, Hu Y (2006). Sca-1+ progenitors derived from embryonic stem cells differentiate into endothelial cells capable of vascular repair after arterial injury.. Arterioscler Thromb Vasc Biol.

[150] Yamamoto K, Sokabe T, Watabe T, Miyazono K, Yamashita JK, Obi S, Ohura N, Matsushita A, Kamiya A, Ando J (2005). Fluid shear stress induces differentiation of Flk-1-positive embryonic stem cells into vascular endothelial cells in vitro.. Am J Physiol Heart Circ Physiol.

[151] Ghazanfari S, Tafazzoli-Shadpour M, Shokrgozar MA (2009). Effects of cyclic stretch on proliferation of mesenchymal stem cells and their differentiation to smooth muscle cells.. Biochem Biophys Res Commun.

[152] Dierick F, Héry T, Hoareau-Coudert B (2016). Resident PW1+ progenitor cells participate in vascular remodeling during pulmonary arterial hypertension.. Circ Res.

[153] Nishimura R, Nishiwaki T, Kawasaki T, Sekine A, Suda R, Urushibara T, Suzuki T, Takayanagi S, Terada J, Sakao S, Tatsumi K (2015). Hypoxia-induced proliferation of tissue-resident endothelial progenitor cells in the lung.. Am J Physiol Lung Cell Mol Physiol.

[154] Spinetti G, Kraenkel N, Emanueli C, Madeddu P (2008). Diabetes and vessel wall remodelling: from mechanistic insights to regenerative therapies.. Cardiovasc Res.

[155] Xie Y, Potter CMF, Le Bras A, Nowak WN, Gu W, Bhaloo SI, Zhang Z, Hu Y, Zhang L, Xu Q (2017). Leptin induces Sca-1+ progenitor cell migration enhancing neointimal lesions in vessel-injury mouse models.. Arterioscler Thromb Vasc Biol.

[156] Schwartz SM, Benditt EP (1977). Aortic endothelial cell replication. I. Effects of age and hypertension in the rat.. Circ Res.

[157] Cheng C, Tempel D, van Haperen R, van der Baan A, Grosveld F, Daemen MJ, Krams R, de Crom R (2006). Atherosclerotic lesion size and vulnerability are determined by patterns of fluid shear stress.. Circulation.

[158] Xu Q (2000). Biomechanical-stress-induced signaling and gene expression in the development of arteriosclerosis.. Trends Cardiovasc Med.

[159] Polacheck WJ, Kutys ML, Yang J, Eyckmans J, Wu Y, Vasavada H, Hirschi KK, Chen CS (2017). A non-canonical Notch complex regulates adherens junctions and vascular barrier function.. Nature.

[160] Zeng L, Xiao Q, Margariti A, Zhang Z, Zampetaki A, Patel S, Capogrossi MC, Hu Y, Xu Q (2006). HDAC3 is crucial in shear-and VEGF-induced stem cell differentiation toward endothelial cells.. J Cell Biol.

[161] Jin ZG, Ueba H, Tanimoto T, Lungu AO, Frame MD, Berk BC (2003). Ligand-independent activation of vascular endothelial growth factor receptor 2 by fluid shear stress regulates activation of endothelial nitric oxide synthase.. Circ Res.

[162] Toledo-Flores D, Schwarz N, Di Bartolo B, Delacroix S, Puranik A, Simari R, Nicholls S, Psaltis P Murine adventitial Sca-1+CD45+ progenitor cells are proangiogenic and give rise to vasa vasorum in atherosclerosis.. Heart Lung Circ.

[163] Le Bras A, Yu B, Issa Bhaloo S, Hong X, Zhang Z, Hu Y, Xu Q (2018). Adventitial Sca1+ cells transduced with ETV2 are committed to the endothelial fate and improve vascular remodeling after injury.. Arterioscler Thromb Vasc Biol.

[164] Owens GK (2007). Molecular control of vascular smooth muscle cell differentiation and phenotypic plasticity.. Novartis Found Symp.

[165] Hu Y, Davison F, Ludewig B, Erdel M, Mayr M, Url M, Dietrich H, Xu Q (2002). Smooth muscle cells in transplant atherosclerotic lesions are originated from recipients, but not bone marrow progenitor cells.. Circulation.

[166] Bentzon JF, Weile C, Sondergaard CS, Hindkjaer J, Kassem M, Falk E (2006). Smooth muscle cells in atherosclerosis originate from the local vessel wall and not circulating progenitor cells in ApoE knockout mice.. Arterioscler Thromb Vasc Biol.

[167] Bentzon JF, Sondergaard CS, Kassem M, Falk E (2007). Smooth muscle cells healing atherosclerotic plaque disruptions are of local, not blood, origin in apolipoprotein E knockout mice.. Circulation.

[168] Iwata H, Manabe I, Fujiu K, Yamamoto T, Takeda N, Eguchi K, Furuya A, Kuro-o M, Sata M, Nagai R (2010). Bone marrow-derived cells contribute to vascular inflammation but do not differentiate into smooth muscle cell lineages.. Circulation.

[169] Roostalu U, Aldeiri B, Albertini A, Humphreys N, Simonsen-Jackson M, Wong JKF, Cossu G (2018). Distinct cellular mechanisms underlie smooth muscle turnover in vascular development and repair.. Circ Res.

[170] Xiao Q, Zeng L, Zhang Z, Hu Y, Xu Q (2007). Stem cell-derived Sca-1+ progenitors differentiate into smooth muscle cells, which is mediated by collagen IV-integrin alpha1/beta1/alphav and PDGF receptor pathways.. Am J Physiol Cell Physiol.

[171] Cruciat CM, Niehrs C (2013). Secreted and transmembrane wnt inhibitors and activators.. Cold Spring Harb Perspect Biol.

[172] Karamariti E, Margariti A, Winkler B, Wang X, Hong X, Baban D, Ragoussis J, Huang Y, Han JD, Wong MM, Sag CM, Shah AM, Hu Y, Xu Q (2013). Smooth muscle cells differentiated from reprogrammed embryonic lung fibroblasts through DKK3 signaling are potent for tissue engineering of vascular grafts.. Circ Res.

[173] Kawano Y, Kypta R (2003). Secreted antagonists of the Wnt signalling pathway.. J Cell Sci.

[174] Wang X, Karamariti E, Simpson R, Wang W, Xu Q (2015). Dickkopf homolog 3 induces stem cell differentiation into smooth muscle lineage via ATF6 signalling.. J Biol Chem.

[175] Margariti A, Winkler B, Karamariti E, Zampetaki A, Tsai TN, Baban D, Ragoussis J, Huang Y, Han JD, Zeng L, Hu Y, Xu Q (2012). Direct reprogramming of fibroblasts into endothelial cells capable of angiogenesis and reendothelialization in tissue-engineered vessels.. Proc Natl Acad Sci USA.

[176] Cheng WL, Yang Y, Zhang XJ, Guo J, Gong J, Gong FH, She ZG, Huang Z, Xia H, Li H (2017). Dickkopf-3 ablation attenuates the development of atherosclerosis in ApoE-deficient mice.. J Am Heart Assoc.

[177] Yu B, Kiechl S, Qi D (2017). A cytokine-like protein dickkopf-related protein 3 is atheroprotective.. Circulation.

[178] Psaltis PJ, Simari RD (2015). Vascular wall progenitor cells in health and disease.. Circ Res.

[179] Nguyen AT, Gomez D, Bell RD (2013). Smooth muscle cell plasticity: fact or fiction?. Circ Res.

[180] Zhang L, Xu Q (2017). Vascular progenitors and smooth muscle cells chicken and egg?. Circ Res.

[181] Chappell J, Harman JL, Narasimhan VM, Yu H, Foote K, Simons BD, Bennett MR, Jørgensen HF (2016). Extensive proliferation of a subset of differentiated, yet plastic, medial vascular smooth muscle cells contributes to neointimal formation in mouse injury and atherosclerosis models.. Circ Res.

